# Asphaltene biotransformation for heavy oil upgradation

**DOI:** 10.1186/s13568-021-01285-7

**Published:** 2021-09-07

**Authors:** Arif Nissar Zargar, Ankur Kumar, Anurag Sinha, Manoj Kumar, Ioannis Skiadas, Saroj Mishra, Preeti Srivastava

**Affiliations:** 1 Department of Biochemical Engineering and Biotechnology, Indian Institute of Technology Delhi, New Delhi, 110016 India; 2 Indian Oil Corporation, R&D Centre, Sector 13, Faridabad, Haryana, India; 3 Department of Chemical Engineering, Technical University of Denmark, Lyngby, Denmark

**Keywords:** Asphaltene, Viscosity, Bacterial consortium, Heavy coil, Reactor

## Abstract

Globally, the reserves of heavy crude oil are seven times more abundant than that of light crude, and yet, they are underutilized because of their high viscosity and density, which is largely due to the presence of large amounts of asphaltenes. Biotransformation of heavy oil asphaltenes into smaller metabolites can be used for reducing their viscosity. Several microorganisms capable of asphaltene biodegradation have been reported but only few have been characterized for its biotransformation. In the present study, a 9-membered microbial consortium was isolated from an oil contaminated soil. About 72% and 75% asphaltene biotransformation was achieved by growing cells at shake flask level and in a 1.5 l bioreactor, respectively. A representative structure of asphaltene was constructed based on LC–MS, ^1^H-NMR, ^13^C-NMR, FT-IR, ICPMS and elemental analysis (CHNS) of *n*-heptane purified asphaltene from Maya crude oil. Biotransformation of asphaltene, as analyzed by performing ^1^H-NMR, FT-IR and elemental analysis, resulted in 80% decrease in S and N when compared to the control along with incorporation of oxygen in the structure of asphaltene. About 91% decrease in the viscosity of the Maya crude oil was observed after two weeks when oil: aqueous phase ratio was 1:9. The results suggest that the isolated microbial consortium can be used for biological upgradation of heavy crude oil. To our knowledge, this is the first report where a microbial consortium resulted in such high asphaltene biotransformation.

**Supplementary Information:**

The online version contains supplementary material available at 10.1186/s13568-021-01285-7.

## Key points

75% Asphaltene biotransformed by a 9 membered microbial consortium.

Decrease in viscosity of theheavy oil was observed.

A decrease in N and S was observed resulting in cleaner fuel.

## Introduction

Depletion of energy sources is a problem of global concern. Out of all the energy sources, the most utilized are the petroleum (32%) and coal (26%). Their limited availability along with their higher consumption rates has led to escalating concerns about their sustainability. Thus, a need is felt to shift to alternative sources of fuel. However, the share of fossil fuels as primary source of energy has only shifted by about 2% in the last 40 years. This slow shift to alternative fuels has made it important to utilize fossil resources in a more efficient way.

Petroleum exists in two forms: conventional and unconventional oils (Leon and Kumar 2005). Unconventional petroleum constitutes about 70% of the total petroleum but has not been utilized due to difficulties in extraction and processing which is because of presence of higher amount of asphaltenes (Lavania et al. 2015). Solubility class definition of asphaltenes defines them as a dark brown to black fraction of the crude oil which is soluble in toluene and insoluble in *n*-alkanes (*n*-heptane and *n*-pentane) (Scotti and Montanari 1998). Structurally, asphaltenes consist of polynuclear aromatic rings, alkyl chains, hetero atoms like (C, N, S and O) and metal ions like (V, Fe, Ni and Zn) which may be arranged either in island pattern or archipelago pattern (Mullins 2011; Ruiz-Morales et al. 2020; Speight and Moschopedis 1982). These asphaltenes lead to higher viscosity and specific gravity of the heavy oil which makes pumping very difficult and therefore increase the total cost of the oil thus produced (Akbarzadeh et al. 2007). During extraction and refining of heavy petroleum, the equilibrium between asphaltenes and resins, which stabilize asphaltenes in crude oil, gets disturbed and leads to precipitation of the asphaltenes (Barth 1962; Uribe‐Alvarez et al. 2011). This precipitated asphalt forms a solid mass which deposits in the pipelines and clogs them (Tavakkoli et al. 2015). Several physical (wireline cutting and hydro blasting), thermal (oil or water vapor injection, heating bottom well) and chemical (solvent or soluble amphiphile injection) methods have been used to remove the precipitated asphaltene (Shahebrahimi et al. 2018). These methods require extreme conditions and are cost and energy intensive.

Biological methods of asphaltene biodegradation or biotransformation offer certain advantages over the ones mentioned above by being specific, less severe, environmentally friendly and cost effective. There are several reports on microbial degradation of asphaltenes (Lavania et al. 2012; Pineda-Flores et al. 2004; Pourfakhraei et al. 2018; Shahebrahimi et al. 2018; Uribe‐Alvarez et al. 2011). During asphaltene biodegradation, microorganisms utilize asphaltene as a primary carbon source for their growth. A few examples of asphaltene biotransformation by either microbes or by their enzymes have also been reported (Ayala et al. 2012; Bertrand et al. 1983; Garcia-Arellano et al. 2004; Mogolloń et al. 1998). During biotransformation, microbes produce enzymes which transform asphaltene into other compounds by either cleaving the aromatic rings or by cutting an internal linkage (C–S bond or C–C bond in aliphatic chain) or by desulfurization, denitrogenation or demetallation. Biotransformation does not remove any carbon from the asphaltene, thereby preserving its calorific value. The amount of asphaltene biotransformed by the reported strains to date are less and the process has been demonstrated at shake flask level only.

Here, we report biotransformation of Maya crude oil asphaltene using a nine membered bacterial consortium isolated by enrichment culture of the oil-contaminated soil. The members were identified by 16S rRNA sequencing. Asphaltene biotransformation was performed in a shake flask as well as in a bench-scale 1.5 l bioreactor. Biotransformation efficiency of 73% was obtained at a shake flask level and 75% at bioreactor level with a maximum asphaltene biotransformation rate of about 8.36 mg/g cells h.

## Materials and methods

### Isolation of microbial consortium

Microbial consortium was isolated by adding asphaltene (2.5 g/l) and 1 g/l of oil-contaminated soil (obtained from Indian Oil Corporation Limited, Faridabad) to minimal salts medium (MSM). The MSM contained (per l): 2 g Na_2_HPO_4_, 1 g KH_2_PO_4_, 3.75 g (NH_4_)_2_C_2_O_4_, 0.4 g MgCl_2_ and was supplemented with filter sterilized trace elements (1 mg/ml) and ammonium sulfate (1000 mg/l). The composition of the trace elements was (per L): 0.05 g KI, 0.05 g LiCl, 0.80 g MnCl_2_.4H_2_O, 0.50 g H_3_BO_3_, 0.10 g ZnCl_2_, 0.10 g CoCl_2_.6H_2_O, 0.10 g NiCl_2_.6H_2_O, 0.05 g BaCl_2_, 0.05 g (NH_4_)_6_Mo_7_O_24_.2H_2_O, 0.50 g SnCl_2_.2H_2_O and 0.10 g Al(OH)_3_. Flasks were set in duplicates and incubated at 30 ºC with shaking at 180 rpm. After 10 days of incubation, dilution plating was performed on MSM agarose (1.5 or 2% w/v) plates overlaid with asphaltene. For this, the MSM medium containing agarose was prepared, autoclaved and poured into sterile culture plates. After the medium solidified, the plates were sprayed with a mixture of asphaltene dissolved in minimum toluene (75 g/l). The plates were left inside the laminar air flow for toluene to evaporate and then dilution plating was performed on the dry asphaltene overlaid MSM plates. Several colonies were observed after 5 days of incubation. Repeated purification rounds, which included growth, followed by serial dilution and plating resulted in isolation of nine different bacterial isolates. Flasks without asphaltene and flasks containing only toluene were used as controls for subculturing and dilution plating.

### Identification of members of the consortium

#### Genomic DNA isolation

Genomic DNA of each member of the consortium was isolated by Genelute Bacterial Genomic DNA kit (Sigma). Lysozyme was used at a concentration of 45 mg/ml to lyse the cells. Wherever necessary, manual genomic DNA isolation method was also used (Marmur 1961).

#### Identification of bacteria

Identification of different members of the consortium was carried out by performing 16S rRNA sequencing of the isolated strains. The16S rDNA of ~ 1.5 kb was amplified using high fidelity Platinum Taq polymerase, genomic DNA as template and primers 16SF and 16SR at an annealing temperature of 58 °C. The PCR product was cloned in pGEMT-easy vector. The clone was confirmed by restriction digestion using EcoRI enzyme (New England Biolabs). For confirmation, sequencing of both the strands was performed. DNA sequencing was carried out at Lifetech Service Laboratory, Invitrogen Bioservices India Private Limited and GCC Biotech (INDIA) Private Limited, India to identify the members of consortium. Maximum likelihood method based on the Hasegawa–Kishino–Yano model was used to construct phylogenetic tree of the isolates (Kishino and Hasegawa 1989).

#### Extraction and purification of asphaltene from Maya crude oil

Asphaltene (referred in following subsections as pure asphaltene) used in the study was obtained from Maya crude oil using ASTM D2007-80 method. Asphaltene was precipitated by adding *n*-heptane (40:1v/v) to the Maya crude oil. The mixture was shaken vigorously and allowed to equilibrate for 2 days after which the precipitated asphaltene was separated by filtering the mixture through 0.22 µm filter. Three rounds of purification of asphaltene were performed by adding *n*-heptane 30 times the volume of maya crude oil taken earlier to dissolve any impurities in *n*-heptane and leave behind pure asphaltene. The precipitated asphaltene was dried in a hot air oven at 40 °C for 48 h. Elemental analysis of this pure asphaltene was performed using vario MACRO cube elemental analyzer.

#### Preparation of the model oil for biotransformation assays

For preparation of the model oil, 4.9 g pure asphaltene was surface sterilized by completely immersing it in 70% ethanol. After evaporating ethanol inside a laminar air flow cabinet, sterilized asphaltene was dissolved in 65 ml toluene (99% pure, Merck) and finally resuspended in 585 ml of *n*-hexadecane (99% pure, ACROS Organics). Both toluene and hexadecane were filter sterilized using a 0.2 µm nylon filter. The model oil prepared was used in subsequent asphaltene biotransformation assays.

#### Method for extraction and estimation of residual asphaltene

In the present study, a method suitable for extraction of pure asphaltene (free from other metabolites) was developed (Additional file 1: Fig. S1). For this purpose, after the completion of the experiment, entire culture broth was transferred into 50 ml falcon tubes and centrifuged at 7000 rpm for 15 min such that the mixture separated into three phases (upper model oil containing asphaltene and trapped biomass, middle aqueous culture medium and a pellet of cells). The three phases were separated by pipetting out the upper two phases into two different falcon tubes. Asphaltene was extracted (from the separated upper phase containing biotransformed model oil and cells trapped with asphaltene) three times with equal volume of toluene. The mixture was centrifuged at 7000 rpm for 15 min at room temperature to pellet down the trapped cells. The liquid phase was pipetted out into a fresh beaker and *n*-heptane in ratio 1:10 was added to the remaining mixture and mixed vigorously for 2 h and finally allowed to settle overnight so that asphaltene alone precipitates. The n-heptane containing some dissolved fractions was pipetted out to leave behind pure asphaltene (free from other metabolites). This asphaltene was again dissolved in 10 ml of toluene and its absorbance was measured in the range of 200–700 nm. Maximum absorbance was obtained at 300 nm which was also found to be concentration dependent. Therefore, quantification of the asphaltene was carried out spectrophotometrically by measuring the absorbance at 300 nm for which a standard curve (in the range of 0.2–16 g/l) was plotted. Estimation of percentage biotransformation of asphaltene was done by comparing the residual asphaltene in test flasks with respect to that of the biotic and abiotic controls (mentioned below). Extraction of asphaltene was also performed from the aqueous phase and the cell pellet after separating upper model oil containing trapped cells, but negligible asphaltene was obtained in this fraction.

From abiotic controls (uninoculated flasks containing media and model oil) and biotic controls (flasks containing media, model oil and dead consortium), both incubated for a period of 3 weeks along with other experimental flasks, out of 150 mg of asphaltene added in each flask, 105.2 mg asphaltene in abiotic controls and 104.8 mg asphaltene in biotic controls was recovered. About 30% of initially supplied asphaltene was lost in all the steps of extraction.

### Asphaltene biotransformation assays

#### Growing cell assay with sucrose or asphaltene as a carbon source

The consortium was prepared by inoculating the microbial strains separately in different test tubes containing 5 ml of Luria broth (LB). The tubes were incubated at 30 ºC, 250 rpm and were harvested when the culture reached an OD_600_ ~ 1. The cells were collected by centrifuging at 5000 rpm for 10 min. The supernatant was discarded, and the pellet was washed with MSM and finally resuspended in 1 ml of fresh MSM. The contents of each tube were mixed to get a bacterial consortium containing each strain in equal number (10^11^ cells/ml). The consortium thus obtained was used to inoculate the Erlenmeyer flasks.

The Erlenmeyer flasks contained 20 ml of the model oil (containing 150 mg asphaltene) added to 60 ml of the MSM supplemented with 17 g/l sucrose (phase ratio of 1:3). It was inoculated with freshly prepared consortium such that the initial optical density at 600 nm (OD_600_) of the aqueous medium was around 0.05. The flasks were placed in a shaking incubator at 30 ºC at 250 rpm for 7 weeks. After 7 weeks, the flasks were harvested and the amount of asphaltene biotransformed with respect to the control (uninoculated biphasic mixture of model oil and aqueous media under similar conditions as sample flasks incubated for 7 weeks) was determined. Growth of the consortium in the inoculated flasks was determined every week by measuring OD_600._ The culture was serially diluted and plated on Luria agar plates to observe the profile of the bacterial colonies.

For monitoring growth on asphaltene as a sole carbon source, experiments were performed as described above, by adding 20 ml of the model oil (containing 150 mg asphaltene) to 60 ml of the MSM except that sucrose was not added in the medium.

#### Growing cell assay with sucrose as carbon source and higher inoculum size

To reduce the total time of the process, a more concentrated inoculum was used. Biphasic medium was prepared by adding 20 ml of the model oil (containing 150 mg asphaltene) to 60 ml of the MSM medium. The flasks were inoculated with higher biomass concentration such that the initial OD_600_ after inoculation was 0.4. The flasks were incubated at 30 ºC at 250 rpm for 3 weeks and the amount of asphaltene biotransformed was determined in each inoculated flask with respect to the control (uninoculated biphasic mixture of model oil and aqueous media under similar conditions as sample flasks incubated for 3 weeks).

#### Resting cell experiment

Metabolically active but non-growing cells (resting cells) were used as biocatalysts. Biphasic mixtures (in triplicate) of model oil and the MSM were prepared and inoculated with the resting cell consortium. For preparation of the resting cell consortium, the primary cultures of different strains were prepared and then used to inoculate flasks containing 300 ml LB. The cultures were incubated at 30 ºC at 250 rpm till OD_600_ ~ 6 was reached. Then the culture was centrifuged at 5000 rpm for 10 min and the pellet was washed with the MSM and the cells stored at – 20 °C. The strains were resuspended in 5 ml of the MSM. The contents of each tube were mixed to get a total of 50 ml of resting cell consortium, which was used to inoculate the flasks containing biphasic mixture of 20 ml of the model oil (containing 150 mg asphaltene) and 60 ml of the MSM to get an initial OD_600_ of 30. Asphaltene biotransformation was carried out at 30 °C for 3 weeks with shaking at 250 rpm. At the end of 7 weeks, the flasks were harvested and the biotransformed asphaltene was measured with respect to the control as described earlier.

#### Control experiments for confirmation of asphaltene biotransformation

Various control experiments were performed in order to confirm biotransformation of asphaltene by the microbial consortium and to determine the sources of various metabolites produced during biotransformation. The details of these control experiments are as follows:

1. Hexadecane biodegradation by the microbial consortium: In this experiment, 20 ml of filer sterilized hexadecane was added to 60 ml of minimal salt medium (MSM) devoid of any carbon source and the flasks were inoculated with the microbial consortium of higher inoculum size. The flasks were incubated for 3 weeks at 30 °C and microbial growth and metabolite formation was determined.
2. Effect of sucrose addition on hexadecane biodegradation: In this experiment, 20 ml filer sterilized hexadecane was added to 60 ml of MSM supplemented with 17 g/l sucrose and the flasks were inoculated with the microbial consortium of higher inoculum size. The flasks were also incubated for 3 weeks at 30 °C and microbial growth and metabolite formation was determined.
3. Microbial biotransformation of crystalline asphaltene: In order to determine if asphaltene can be biodegraded when supplied in higher amounts to support biomass growth, pure sterilized crystalline asphaltene was added (11.25 g/l) to the aqueous medium. The amount of asphaltene added to the flasks was calculated by performing the carbon balance of asphaltene with 17 g/l sucrose. The flasks were again inoculated with the microbial consortium of higher inoculum size and then incubated for 3 weeks at 30 °C during which microbial growth and metabolite formation was determined.
4. Asphaltene biotransformation experiment in which ammonium oxalate was replaced by ammonium chloride in MSM supplemented with sucrose: In order to determine the metabolites arising from ammonium oxalate, MSM containing 17 g/l sucrose and 3.63 g/l ammonium chloride devoid of ammonium oxalate was inoculated with microbial consortium of higher inoculum size. The flasks were incubated for 3 weeks at 30 °C during which microbial growth and metabolite formation was determined.
5. Effect of dead biomass on extraction of residual asphaltene. A biotic control was set up for determining the efficiency of extraction. This served to determine the loss of asphaltene attached to the biomass during the extraction process which could result in inaccurate determination of asphaltene biotransformation. For this, the dead biomass was added to the flask containing model oil and aqueous medium such that the OD_600_ of the flask was ~ 14.5. The flasks were incubated for 3 weeks at 30 °C during which microbial growth and metabolite formation was determined. At the end of the experiment, residual asphaltene was extracted from the flasks and compared with that of the abiotic control (uninoculated flasks containing the model oil and the aqueous medium). The microbial consortium was killed by immersing it in 70% ethanol for 24 h and then drying it in oven at 50 °C.
6. Asphaltene biotransformation with ammonium oxalate as carbon source: In order to evaluate the contribution of ammonium oxalate to growth of the microbial consortium and possible biotransformation of asphaltene, 60 ml of MSM devoid of any carbon source was supplemented with 4.25 g/l ammonium oxalate and 20 ml of model oil was added to the flasks. The flasks were incubated for 3 weeks at 30 °C during which microbial growth and metabolite formation was determined.
7. Heptamethylnonane utilization by microbial consortium: as a control, a non-toxic, non-biodegradable aliphatic carrier phase, such as, heptamethylnonane (HMN) was also tested to identify the contribution of hexadecane to appearance of metabolites during asphaltene biotransformation. For this, 20 ml of filtered HMN was added to 60 ml of the MSM devoid of any carbon source and the flasks were inoculated with the microbial consortium of higher inoculum size (control set 7a). The flasks were incubated for 3 weeks at 30 °C and microbial growth and metabolite formation was determined. Since sucrose was added initially as a carbon source, it was decided to evaluate the effect of sucrose addition on HMN utilization. For this, 20 ml of the filer sterilized HMN was added to 60 ml of MSM, supplemented with 17 g/l sucrose (control set 7b) and the flasks were inoculated with the microbial consortium of higher inoculum size. The flasks were then incubated for 3 weeks at 30 °C and microbial growth and metabolite formation was determined.
8. Asphaltene biotransformation experiment using HMN as inert carrier phase and media supplemented with sucrose: In this experiment, model oil was prepared by dissolving 150 mg of pure sterilized asphaltene in 2 ml of filtered toluene and resuspending the mixture in 18 ml of filtered HMN. Model oil (20 ml) was added to 60 ml of MSM supplemented with 17 g/l sucrose and inoculated with microbial consortium of higher inoculum size. The flasks were incubated for 3 weeks at 30 °C and microbial growth and metabolite formation was determined.

#### Asphaltene biotransformation in a 1.5 l bench-scale bioreactor

Asphaltene biotransformation was also carried out under controlled conditions in a stirred tank (Multifors 2) bench top reactor with a vessel diameter of 8.6 cm and a working volume of 1 l, equipped with two Rushton turbine impellers of diameter 38 mm. The stirrer speed was chosen such that efficient mixing of the two phases (model oil and the aqueous medium) was achieved so as to ensure that the phase ratio of oil and the aqueous medium remained constant even after multiple sampling. This was ensured by mixing the two phases at different stirrer speeds and choosing the speed at which the withdrawal of sample resulted in a sampling phase ratio of 1:3, i.e. same as that of the reactor. After choosing the stirrer speed, the operational flow rate of air was calculated so as to ensure that efficient gas dispersion was achieved and the impeller flooding was prevented. The equations used to calculate the operational gas flow rate are:

Froude number:

1 $$(Fr)=\left(\frac{{N}_{i}^{2}{D}_{i}}{g}\right)$$

For complete gas dispersion:

2 $$F{l}_{g}=0.2{\left(\frac{{D}_{i}}{{D}_{T}}\right)}^{0.5}{\left(Fr\right)}^{0.5}$$

Maximum gas flow rate:

3 $${F}_{g}=\left(\frac{F{l}_{g}}{{N}_{i}{D}_{i}^{3}}\right)$$

where Fr is the Froude number which is the ratio of the flow inertia to the external field, usually gravity, N_i_ is the stirrer speed, D_i_ is the impeller diameter, g is the acceleration due to gravity, Fl_g_ is the gas flow number for complete gas dispersion, D_T_ is the tank diameter and F_g_ is the operational gas flow rate calculated.

Kinetic studies of asphaltene biotransformation were performed after ensuring proper mixing and the complete gas dispersion in the reactor. Two bioreactors were run in parallel and 750 ml of the aqueous medium, as mentioned above, was prepared for each bioreactor. Sucrose (at 17 g/l) was used as a carbon source and ammonium oxalate (at 4.25 g/l) was used as a nitrogen source. After adding the medium to the bioreactors, the component parts of the reactor were mounted on it and the entire set-up was sterilized by autoclaving it at 121 °C for 15 min. The pO_2_ was calibrated between 100% (using air) and 0% (using nitrogen). Sterile model oil (250 ml model oil containing 1.87 g asphaltene containing hexadecane) was added to the reactor using autoclaved tubing such that the total volume of the liquid inside the reactor was 1 l. The pO_2_ was again calibrated to 100% after addition of the model oil. The consortium was prepared and 40 ml was added to the reactors such that the initial OD_600_ after inoculation was ⁓ 0.4. The reactors were operated at 30 °C and pH 7 for a period of 3 weeks with 1 sampling per day for the first week and then 1 sampling every alternate day. For controlling the pH, 1 M HCl and NaOH solutions were prepared and connected to the reactors. The volume of the sample withdrawn each time was 8 ml (containing 2 ml of the model oil and 6 ml of the aqueous medium). From every sample, biomass growth was estimated by taking the OD_600_ of the aqueous broth. Amount of asphaltene biotransformed was determined by extraction of asphaltene from the model oil phase and comparing it with the control (sample taken from the uninoculated reactor operated under similar conditions run and in parallel for 3 weeks). Carbon source utilization and metabolite formation during biotransformation were estimated by performing HPLC of the aqueous phase. After 3 weeks, the batch runs was stopped and the total asphaltene biotransformed was determined with respect to the control (asphaltene extracted from an uninoculated reactor).

For detection of sugars, HPLC was performed using Thermo Scientific™ Dionex™ ICS-6000 equipped with Dionex ICS-6000 CD conductivity detector. Samples were diluted 100 times in milli-Q water and 20 µl of the sample was injected into the column. About 10 mM NaOH was used as the eluent at a flow rate of 0.5 ml/min. Volatile fatty acids (VFA) and alcohols were determined using an HPLC (Shimadzu, USA) equipped with a refractive index detector and an Aminex HPX-87H column (Bio-Rad, USA) at 60 °C. A solution of 12 mM H_2_SO_4_ was used as an eluent at a flow rate of 0.6 ml/min. For detection of sugars on Dionex system, standards of HPLC grade sucrose, glucose and fructose were prepared in the range 0.01–1 g/l and on Dionex system these sugars were detected at retention time of 10.35, 9.93 and 11.43 min. For detection of sugars, sugar acids and alcohols on Shimadzu system, standards for sugars (glucose, fructose, and sucrose), acids (lactic acid, valeric acid, caproic acid, formic acid, acetic acid and propanoic acid) and alcohols (ethanol, propanol and propanediol) were prepared in the range 0.1 to 5 g/l. Glucose, fructose and sucrose showed a retention time of 8.7, 9.4 and 8.5 min, respectively. Lactic acid, valeric acid, caproic acid, formic acid and propanoic acid showed a retention time of 12.32, 27.88, 41.40, 13.76, 17.25 min, respectively. Ethanol, propanediol and glycerol had a retention time of 21.605, 17.96 and 12.98 min, respectively.

To confirm production of acid and alcohol during asphaltene biotransformation, 0.5 ml of the aqueous samples collected during each sampling was taken and its GCMS was performed by injecting 10 μl of sample into the column. For identification of the sample, ions were analyzed and identified using 5977B GC-MSD, gas chromatography mass spectrometer (Agilent). The injection temperature was 260 °C and the oven temperature was programmed to start at 40 °C, (held for 2 min and then ramped to 280 °C at a rate of 6 °C /min) and finally to 280 °C (at a rate of 10 °C/min).

#### Enzyme production during asphaltene biotransformation

Culture broth collected during the asphaltene biotransformation experiment was used for assaying the production of various extracellular enzymes during the course of experiment. The culture broth was taken and centrifuged at 5000 rpm for 20 min at 4 °C to pellet down the cells. The cell free supernatant was used for determining the production of various secreted enzymes which include dioxygenases (catechol 1,2 dioxygenase and catechol 2,3 dioxygenase), laccases and peroxidases. The pellet containing cells was used for assaying production of monooxygenases. In all these experiments, uninoculated medium was used as the control.

For assaying the production of dioxygenases during asphaltene biotransformation, catechol 1,2 dioxygenase and catechol 2,3 dioxygenase assay were performed as described by Silva et al. (2012). Indole-indigo test was used for assaying the production of monooxygenases following the previously described method (O'Connor et al. 1997). Measurement of laccase activity was performed according to Eggert et al. (1996). Manganese peroxidase (MnP) assay was performed as described by Wariishi et al. in 1992 (Wariishi et al. 1992). Lignin peroxidase assay was performed with veratryl alcohol (VA) as a substrate as described (Camarero et al. 1999).

### Representative structure of asphaltene

For chemical characterization and prediction of the structure of asphaltene, LC–MS, ^1^H-NMR, ^13^C-NMR, FT-IR, CHNS and ICPMS analysis of *n*-heptane purified asphaltene was performed and the data obtained from all was used to construct a representative structure of asphaltene.

LC–MS was performed using LC–MS Agilent Technologies 1260 Infinity LC and 6410 Triple Quad MS, USA equipped with WATERS ACQUITY QSM pump and WATERS ACQUITY FTN autosampler. LC–MS was performed in a lock spray configuration with a reference scan frequency of 10 s, reference con voltage of 40 V and capillary voltage of 3 kV. Instrument was calibrated and calibration parameters were set as follows: Lteff: 1800, Veff: 7217, resolution: 18,000 and trigger threshold: 1.40 V. Five mg of pure asphaltene was dissolved in 1 ml of toluene and 2 µl sample was injected into the column at a flowrate of 0.1 ml/min. Experimental instrument parameters were set as follows: polarity: ES positive, capillary voltage: 3 kV, source temperature: 80 °C, desolvation temperature: 300 °C, desolvation gas flow rate: 7000 L per hour and trap gas flow rate: 0.40 ml/min. Scanning of ranges from 100 to 1200 with a peak detection window of 1 was performed during detection time of 20 min (Riedeman et al. 2016). Ions were surveyed in positive ES mode and the *m*/*z* values obtained were analyzed using METLIN.

The ^1^H and ^13^C-NMR of purified asphaltene was carried out using Bruker Avance 800 spectrometer operating at 14.09 T. For ^1^H-NMR, 25 mg of *n*-heptane purified asphaltene was dissolved in 1 ml of CdCl_3_ while for ^13^C-NMR, 250 mg of purified asphaltene was dissolved in 1 ml of CdCl_3_. TMS was used as internal standard. The chemical shift obtained at 77 ppm in ^13^C-NMR and 0 ppm in ^1^H-NMR was referred to as central signal of CdCl_3_. For ^1^H-NMR, spectra were acquired with spectral width of 8.01 kHz, pulse angle of 90° (14 µs) and a delay time of 1 s. For ^13^C-NMR, the spectra were acquired with spectral width of 24.04 kHz, pulse angle of 30° (9.5 µs) and a delay time of 2 s.

FT-IR spectroscopy of extracted asphaltene was performed to determine the functional groups and the type of bonding present in the asphaltene. FT-IR spectroscopy was done using FT-IR Thermo Fisher Scientific Nicolet iS50 in ATR mode considering a range of 400–4000 cm^−1^.

Elemental analysis (CHNS) of the *n*-heptane purified asphaltene was done using Elementar vario el cube elemental analyzer. Five milligrams of pure asphaltene were weighed on a precision scale and placed in the tin capsule and inserted into the carousel. The contents were burned in a combustion chamber set at 1150 °C. The reduction chamber was set at 850 °C. The analysis was performed in triplicates and the elemental percentage was determined by mass difference.

The *m*/*z* values obtained from LC–MS were analyzed using Metlin and the library of the hits was obtained. Primary screening of these hits was done based on the FT-IR data. The hits of the LC–MS data with bonding and functional groups different from the ones detected by the FT-IR were discarded and those with similar functional groups and bonding were further screened based on H^1^ and ^13^C-NMR data obtained. Finally, the shortlisted compounds were used to construct a representative structure of asphaltene using Chemdraw software.

### Structural and chemical changes in asphaltene due to biotransformation

To gain an insight into the structural and chemical changes in asphaltene due to microbial biotransformation, asphaltene was extracted and purified from the experimental set-ups. FT-IR, ^1^H-NMR, ^13^C-NMR and CHNS were performed. The data obtained from these were compared to those obtained with the appropriate controls (pure asphaltene and asphaltene harvested from the uninoculated flasks). The protocol followed for FT-IR, ^1^H-NMR, ^13^C-NMR and CHNS was the same as described above.

### Biosurfactant production by members of the consortium

Individual members of the consortium were screened for the production of biosurfactants. Microbial strains were inoculated separately in LB and incubated for a period of 7 days. After completion of incubation, the cultures were harvested by centrifuging at 7000 rpm for 20 min at 4 °C. The cell free supernatants were used for assaying the production of biosurfactants using the drop collapse assay, emulsion index assay, oil displacement assay and microplate as described by various groups (Cooper and Goldenberg 1987; Ghomi et al. 2012; Jain et al. 1991; Morikawa et al. 2000; Persson and Molin 1987). Uninoculated medium was used as a negative control and 10% SDS solution was used as a positive control. To confirm the production of the biosurfactant by individual members of the consortium, the strains were cultured separately and reduction in the surface tension of the medium due to growth of individual members was measured. For measurement of surface tension, a digital K12 Kruss tensiometer was used and measurements were performed using Du Noüy ring method. Accuracy of the tensiometer was checked by measuring the surface tension of double distilled water. Uninoculated medium incubated over a period of 7 days was used as a negative control in the experiment.

### Effect of biosurfactant on asphaltene present in model oil

To determine the effect of the biosurfactant on asphaltene, an experiment was set in which all the biosurfactant producing strains were cultured for 7 days in 100 ml of MSM supplemented with sucrose (medium used for asphaltene biotransformation studies). After incubation the cultures were harvested by centrifuging the culture broth at 7000 rpm for 20 min at 4 °C. Biosurfactants were extracted from the supernatant according to method described by Xu et al. (2020). The biosurfactants were mixed together, weighed and added to 20 ml of the model oil containing asphaltene dissolved in toluene and resuspended in hexadecane. The contents in the flask were incubated at 30 °C for 3 weeks at 250 rpm. At the end of incubation, asphaltene was harvested from the flasks as described earlier (see “Materials and methods”) and FTIR and NMR analysis of the harvested asphaltene was performed to determine any change in the structure due to addition of biosurfactant. Flasks containing only model oil (without any biosurfactant) served as negative control and control for determining the asphaltene biotransformed while flasks in which tween 80 of weight equal to biosurfactant mixture added in other flasks served as positive control.

### Upgradation of heavy Maya crude oil

To determine the efficiency of the microbial consortium in upgrading heavy crude oil, 10 ml of Maya crude oil was added to 90 ml of aqueous medium. The composition of the medium was the same as that used in asphaltene biotransformation experiments. The flasks were inoculated with microbial consortium of higher inoculum size such that the OD_600_ of the aqueous phase after inoculation was 0.4. The flasks were incubated at 30 °C at 250 rpm for 14 days. Uninoculated flasks containing 10 ml of the Maya crude oil and 90 ml aqueous medium served as the controls. At the end of incubation, the flasks were harvested, the oil and the aqueous phase were separated by centrifugation at 7000 rpm for 15 min. The oil phase was transferred to a fresh tube and its viscosity was measured and changes in elemental composition were determined. From the collected oil, asphaltenes were extracted as described earlier and amount of asphaltene biotransformed (with respect to control) during oil upgradation was determined.

Viscosity measurements of oil were performed using Anton paar MCR 302 rheometer (Peralta-Martínez et al. 2011). The configuration used for determination of the viscosity was the parallel plate method. For this, 1 ml of the cell free oil was placed between the plates and it was sheared between 0 to 200 s^−1^. The viscosity of the oil was measured at the shear rate of 200 s^−1^. Various controls used for viscosity measurements were MilliQ water, hexadecane, glycerol, toluene, Roncador crude oil and Usan crude oil.

Elemental analyses of the oil were performed by taking 0.05 ml of cell free oil in tin drum and placing it in carousal of the elemental analyzer and performing the analysis as described earlier with asphaltene.

## Results

### Isolation of the microbial consortium

A 9 membered microbial consortium was isolated from the MSM plates containing asphaltene. Based on the16S rDNA sequence, the members of the consortium were identified as *Arthrobacter* sp.1 IITD 100, *Rhodococcus* sp. IITD 101, *Arthrobacter* sp.2 IITD 102, *Barrientosiimonas* sp. IITD 103, *Lysinibacillus* sp. IITD 104, *Sporosarcina* sp. IITD 105, *Bacillus* sp. IITD 106, *Micrococcus* sp. IITD 107 and *Paenibacillus* sp. IITD 108. The 16S rDNA sequence was submitted to GenBank and the accession numbers assigned are given in Table 1. The strains were submitted to Microbial Type Culture Collection and Gene Bank, Chandigarh and have been allotted the accession numbers MTCC 25271, MTCC 25272, MTCC 25273, MTCC 25274, MTCC 25275, MTCC 25276, MTCC 25277, MTCC 25278 and MTCC 25279, respectively (Table 1).

**Table 1:** Details of strains of the consortium

S. No.	Name of bacterium	MTCC number	Genbank accession number	Genus reported for PAH/asphaltene degradation
1	*Arthrobacter* sp.1	MTCC 25271	MT830848	Polycyclic alkane, aromatic hydrocarbon and asphaltene degradation
2	*Arthrobacter* sp.2	MTCC 25272	MT830850	Petroleum hydrocarbon and asphaltene degradation
3	*Rhodococcus* sp.	MTCC 25,273	MT830849	Crude oil and aromatic hydrocarbon degradation
4	*Barrientosiimonas* sp.	MTCC 25274	MT830851	No reports
5	*Lysinibacillus* sp.	MTCC 25275	MT830852	PAH and asphaltene degradation
6	*Sporosarcina* sp.	MTCC 25276	MT830853	PAH degradation
7	*Bacillus* sp.	MTCC 25277	MT830854	PAH and asphaltene degradation
8	*Micrococcus* sp.	MTCC 25278	MT830855	PAH and asphaltene degradation
9	*Paenibacillus* sp.	MTCC 25279	MT830856	PAH degradation

### Asphaltene biotransformation by the identified consortium

ASTM D2007-80 method was used with the Maya crude oil and resulted in separation of dark black colored crystalline asphaltene. Three rounds of purification resulted in extraction of pure asphaltene which was found to be soluble in toluene and insoluble in *n*-heptane. Elemental analysis of this fraction showed that it contained 78–81 weight % carbon, 8.5–8.9 weight % hydrogen, 0.25–0.29 weight % sulfur and 2.7–3.1 weight % nitrogen.

**Fig. 1:**
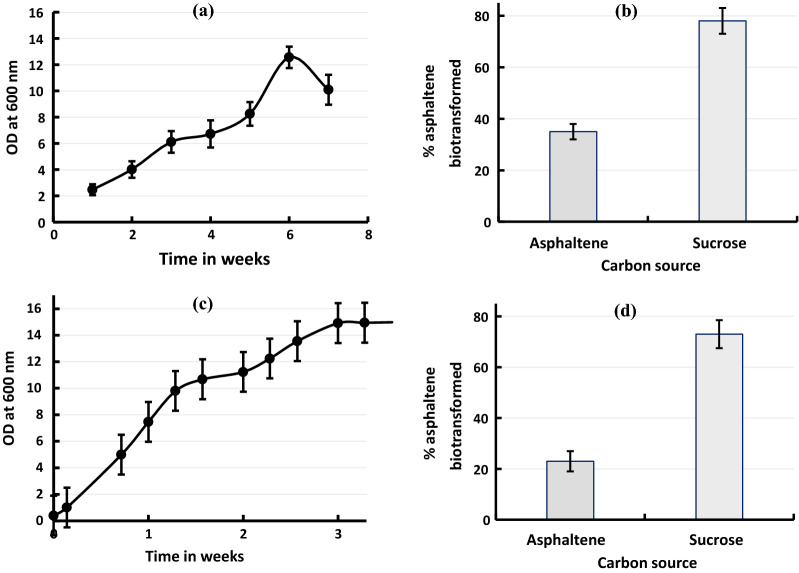
Asphaltene biotransformation at shake flask level. **a** Growth profile of consortium in seven weeks growing cell experiment in the presence of both asphaltene and sucrose where the latter is used as primary carbon source. **b** Percentage asphaltene biotransformation by consortium with respect to controls in seven weeks growing cell experiment. The left bar represents asphaltene alone and the right bar represents asphaltene and sucrose where the latter serves as primary carbon sources. **c** Growth profile of consortium in 3 weeks growing cell experiment with higher inoculum size in the presence of both asphaltene and sucrose where the latter is used as primary carbon source. **d** Percentage asphaltene biotransformation by consortium with respect to controls in three weeks growing cell experiment with higher inoculum. The left bar represents asphaltene alone and the right bar represents asphaltene and sucrose where the latter serves as primary carbon sources

Growing cell experiment with sucrose as a carbon source and initial OD_600_ ~ 0.05 revealed maximum asphaltene biotransformation of 78% after 7 weeks. The concentration of the asphaltene used was 2.50 g/l and the amount left after 7 weeks was 0.55 g/l. A diauxic growth pattern was obtained in which initial increase in cell number was seen till 3 weeks followed by a lag (to week 4) and then a secondary log phase from week 5 to week 6 after which a death phase occurred (Fig. 1a). The secondary lag obtained near week 4 could be due to exhaustion of sucrose in the medium and adaptation of the bacteria to hexadecane as a secondary carbon source. No growth was obtained in the uninoculated flasks containing the biphasic medium. These served as controls for determining the amount of asphaltene biotransformed by the consortium. Growing cell experiment with asphaltene as a sole source of carbon and initial OD_600_ ~ 0.05 revealed average asphaltene biotransformation of 35% after 7 weeks. The concentration of asphaltene added at the start of the experiment was 2.50 g/l and the amount left after 7 weeks was 1.63 g/l (Fig. 1b). Growth was observed till 4 weeks after which the cell concentration declined (Additional file 1: Fig. S2a).

Use of higher inoculum resulted in a maximum asphaltene biotransformation of 73% when sucrose was used as a primary carbon source. However, only 20–25% asphaltene was utilized in 3 weeks when asphaltene was used as a sole carbon source (Fig. 1c, d). With resting cells, maximum asphaltene biotransformation of 25–30% was achieved in 3 weeks (Additional file 1: Fig. S2b). The experiments with higher inoculum size were incubated only for 3 weeks as no further biomass growth was observed because the cells had entered stationary phase.

### Control experiments for confirmation of asphaltene biotransformation

The results of the control experiments are presented in Table 2. The consortium was found to be capable of utilizing hexadecane as a carbon source (control exp 1) however the maximum OD_600_ on hexadecane was 2.9 (Additional file 1: Fig. S3a) which was very low when compared to the experimental set in which the aqueous phase was supplemented with sucrose (control exp 2) (Additional file 1: Fig. S3c). Microbial consortium was found to be incapable of efficiently utilizing crystalline asphaltene as a carbon source for its growth (Additional file 1: Fig. S5e) (control exp 3). Maximum biomass growth corresponding to OD_600_ value 2.34 was obtained when 11.25 g/l crystalline asphaltene was used in the experiment. In asphaltene biotransformation experiments where in model oil was used, total asphaltene concentration of 1.5 g/l was used in the experiments, therefore biomass growth in those experiments could not be due to utilization of asphaltene. In the experimental set where ammonium oxalate was replaced with ammonium chloride (control exp 4), no significant variations in growth or metabolite production (Additional file 1: Fig. S4a) were observed form normal asphaltene biotransformation experiment. This suggested that ammonium oxalate at the concentration used did not promote microbial growth or asphaltene biotransformation. In control experiment 5, in which effect of dead biomass on asphaltene extraction was evaluated, the amount of asphaltene recovered from the flask was 104.8 mg in comparison to 105.2 mg extracted from the abiotic control (uninoculated flask containing model oil and aqueous medium). This confirmed that there was negligible loss of asphaltene due to attachment to biomass. Around 30% of asphaltene lost in both biotic and abiotic control occurred during various steps of extraction. In the control experiment 6, in which ammonium oxalate was used as a carbon source, the biomass growth (Additional file 1: Fig. S4c) and asphaltene biotransformation was attributed to the presence of hexadecane in the flasks. It was inferred that at the concentration used in the experiments, there is no significant effect of ammonium oxalate on biomass growth and asphaltene biotransformation. From control experiment 7a, it was inferred that the microbial consortium was incapable of utilizing HMN as no growth and metabolite formation was observed in this set of experiment. When model oil was prepared using HMN as a carrier phase and the medium was supplemented with sucrose (control set 8), significant microbial growth (Additional file 1: Fig. S5a) and asphaltene biotransformation (70.35%) occurred. No diauxic growth was obtained which confirmed that the diauxic growth pattern observed in other experiments was because of secondary growth of the microbes on hexadecane.

**Table 2:** Control experiments for asphaltene biotransformation

Experiment	Control set	Composition	Maximum Biomass growth (OD_600_)	Percentage Asphaltene biotransformed
Controls for determination of asphaltene biotransformation	Abiotic control	Model oil (asphaltene in hexadecane or HMN) and MSM supplemented with sucrose but uninoculated	0	0.00
Asphaltene biotransformation without sucrose		MSM without sucrose, Asphaltene in hexadecane and Microbial consortium	2	28.57
Effect of sucrose addition on biotransformation of asphaltene		MSM with sucrose, Asphaltene in hexadecane and Microbial consortium	12.32	72.64
Hexadecane biodegradation by the microbial consortium	1	MSM without sucrose, Hexadecane and Microbial consortium	2.92	NA
Effect of sucrose addition on hexadecane biodegradation	2	MSM supplemented with sucrose, Hexadecane and Microbial consortium	12.56	NA
Microbial biotransformation of crystalline asphaltene	3	MSM without sucrose, 11.25 g/l crystalline asphaltene and microbial consortium	2.34	7.65
Asphaltene biotransformation experiment in which ammonium oxalate was replaced by ammonium chloride in MSM and supplemented with sucrose	4	MSM containing ammonium chloride and supplemented with sucrose, Asphaltene in hexadecane and Microbial consortium	10.33	69.13
Effect of dead biomass on extraction of residual asphaltene	5 Biotic control	MSM without sucrose, Asphaltene in hexadecane and dead microbial consortium	14.52	0.35
Asphaltene biotransformation experiment with ammonium oxalate as carbon source	6	MSM containing ammonium oxalate but no sucrose, Asphaltene in hexadecane and Microbial consortium	2.21	28.54
Heptamethylnonane utilization by microbial consortium	7a	MSM without sucrose, Heptamethylnonane and Microbial consortium	0.64	NA
Effect of sucrose addition on HMN utilization	7b	MSM with sucrose, Heptamethylnonane and Microbial consortium	8.97	NA
Asphaltene biotransformation experiment using HMN as inert carrier phase and media supplemented with sucrose	8	MSM supplemented with sucrose, Asphaltene in Heptamethylnonane and Microbial consortium	8.86	70.35

All the control experiments were performed with higher inoculum size (initial OD_600_ value 0.4) therefore, the comparison has to be made with higher inoculum asphaltene biotransformation experiment.

From HPLC and GCMS studies of the aqueous phase, it was found that most of the metabolites detected in the reactor studies were obtained only when sucrose was added to the flasks. It is likely that the acids and the alcohols produced (Additional file 1: Fig. S3–S5) are the result of metabolization of sucrose by the microbial consortium.

### Representative structure of asphaltene

To determine the structural changes in asphaltene as a result of biotransformation, it was necessary to determine its structure in the native state. The *m*/*z* values obtained from the LC–MS were analyzed using METLIN and a search performed at an accuracy of 10%, indicated presence of 3687 different compounds. FT-IR spectra showed the presence of various functional groups in the structure of asphaltene. Strong and narrow peaks obtained at 1370 and 1450 cm^−1^ corresponding to C–H bending showed the presence of various alkanes (Isopropyl, methyl and methylene). Small peak corresponding to C=C stretching was obtained at 1600 cm^−1^, which showed the presence of aromatic or C=C stretching alkenes. C–H stretching, alkanes or CH_2_ symmetric vibrations were also detected at 2850 and 2920 cm^−1^. Small peaks corresponding to O–H stretching, alcohols and phenols were also detected at 3600 and 3620 cm^−1^. Peaks corresponding to N–H and C–S were detected at 3350 cm^−1^ and 721 cm^−1^, respectively.

In ^1^H-NMR spectra, several peaks for saturated alkanes were detected. Chemical shifts at 0.1, 0.8, 1.0 and 1.2 showed the presence of hydrogens present in R–H, RC–H, RO–H and R_2_N–H hydrogens, respectively. Chemical shifts at 2.0 and 2.17 correspond to hydrogens present in R_2_N–CH, NC–CH, RCOO–H, R_2_C=CR–CH. Hydrogens present in RS–CH and R_2_–NH were detected as chemical shifts at 2.3 ppm. Chemical shift obtained at 7.1 and 7.2 ppm corresponded to hydrogens of Ph–OH, RCON–H and other aromatics.

In ^13^C-NMR spectra, various chemical shifts for carbon atoms in saturated alkanes were detected in the range 0.10 to 39.40 ppm. Chemical shifts in the range of 76 and 77 ppm correspond to carbons attached to heteroatoms like C–OH, C–OR, C–N and C–S. Chemical shifts at 125 and 131 ppm corresponded to their presence in aromatic compounds. Screening on the basis of FT-IR, ^1^H-NMR and ^13^C-NMR data resulted in elimination of 3678 compounds out of the previously selected 3687 compounds. Final nine compounds selected for constructing a representative structure of asphaltene included (Tetra-butyl, phenyl, methylene) dibenzene, Dibenzotetracene, Thiooxane, Methyl–Napthochrysene, Dibenzochrysene, Anthracenedione, Dibenzothiophene, Carbazole and Methyl, nitrophenyl fluorene. A representative structure was constructed using Chemdraw by linking these compounds using small alkyl chains (Additional file 1: Fig. S6). Elemental analysis of the pure asphaltene showed that the weight fraction of C, H, N and S was 80.85%, 8.9%, 3.18% and 0.357%, respectively. The weight fractions of the elements in the constructed structure were calculated and were found to be similar to the values determined by the elemental analyzer.

### Changes in the composition of asphaltene due to biotransformation

**Fig. 2:**
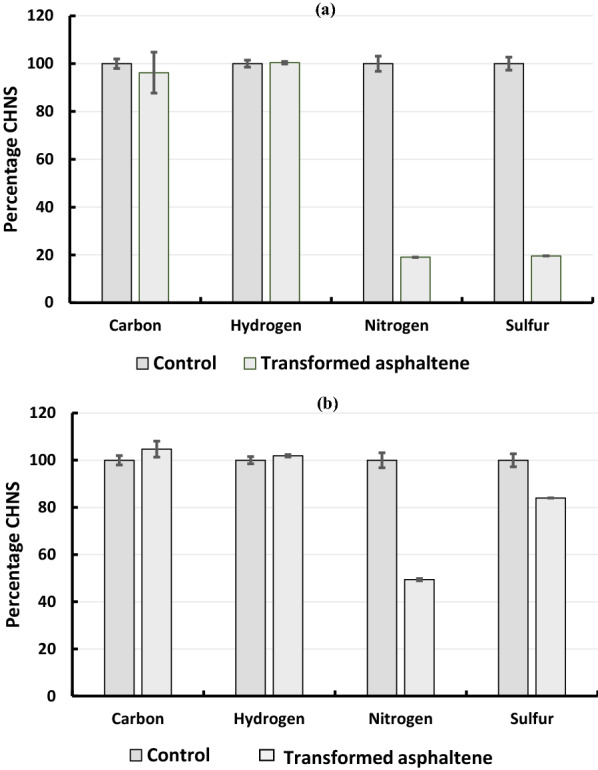
Elemental analysis of untreated and biotransformed asphaltene. **a** Elemental changes in asphaltene due to biotransformation by growing consortium. **b** Elemental changes in asphaltene due to biotransformation by resting cells

Elemental changes in asphaltene during transformation by growing cells revealed no major changes in the C and H content while about 80% decrease was observed in the N and S content. This suggested that the consortium biotransformed asphaltenes without causing much change in its calorific value (Fig. 2a). Asphaltene biotransformation by resting cells also did not reveal any changes in the C and H content but a 45% decrease in N and 18% decrease in S was observed. No decrease in C and H was observed (Fig. 2b).

**Fig. 3:**
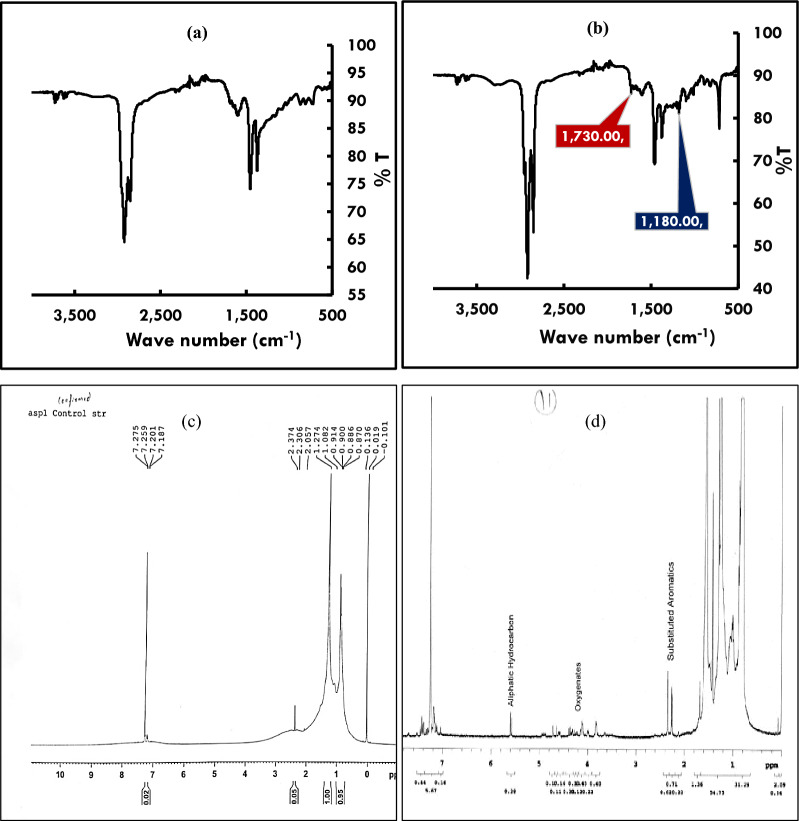
Changes in the composition of asphaltene due to biotransformation. **a** FT-IR spectrum of the control asphaltene. **b** FT-IR spectrum of the biotransformed asphaltene showing incorporation of oxygen in the structure of asphaltene. **c** ^1^H-NMR spectrum (zoomed) of control asphaltene. **d** ^1^H-NMR spectrum (zoomed) of the biotransformed asphaltene showing disappearance of sulfur, nitrogen and formation of oxygenates in the structure of asphaltene due to biotransformation

FT-IR results revealed incorporation of oxygen into asphaltene as ether and aldehyde, ketone or carboxylic acid group. Two peaks, one at 1730 cm^−1^ and another at 1180 cm^−1^ were observed in the treated sample which corresponded to C=O stretching and C–O–C ether bond stretching respectively. These were found to be missing in the untreated controls (Fig. 3a, b). This suggested that oxygen was inserted into the asphaltene and pointed to involvement of oxygenases.

NMR studies of the biotransformed asphaltene revealed reduction in sulfides, N–C bonds and formation of oxygenates. The results of the FT-IR and CHNS were confirmed by the NMR data of control and the treated asphaltene. The disappearance of peak at 2.2 ppm due to biotransformation suggested removal of N and S from asphaltene. Appearance of peaks from 3.5 ppm to 4.5 ppm revealed formation of oxygenates as a result of biotransformation (Fig. 3c, d).

### Asphaltene biotransformation in a 1.5 l bioreactor

Asphaltene biotransformation was also performed on a larger scale in a 1.5 l bioreactor. For achieving good mixing between the oil and the aqueous phase, the biphasic mixture was stirred at different rpm (200, 250, 300, 350, 400, 450 and 500). Complete mixing of the two phases (i.e., sample withdrawn from the reactor-maintained phase ratio 1:3 which was the phase ratio of model oil and aqueous media inside the reactor) was observed at stirrer speed of 350 rpm. So, 350 rpm was chosen as the operational stirrer speed. Operational gas flow rate into the reactor was calculated by first calculating the Froude number and then the gas flow number and finally the gas flow rate. Froude number was calculated using Eq. (1) and was equal to 0.140. Gas flow number for complete gas dispersion was calculated using Eq. (2) and was equal to 0.0497. Maximum gas flow rate to prevent impeller flooding and loading and to ensure complete gas dispersion was calculated using Eq. (3) and was found to be 0.95 l min^−1^. The operational gas flow rate into the system was chosen to be 0.9 l min^−1^. Impeller Reynolds number was calculated to be equal to 10,562.7 (> 4000), which ensured perfect turbulent flow of the liquid inside the reactor. The K_L_a for both the reactors were determined experimentally using the dynamic method and was found to be 0.17 s^−1^.

The addition of model oil to the reactor, decreased the pO_2_ from 100 to 85%, therefore the pO_2_ was again calibrated to 100% after addition of the model oil to the fermentation medium.

**Fig. 4:**
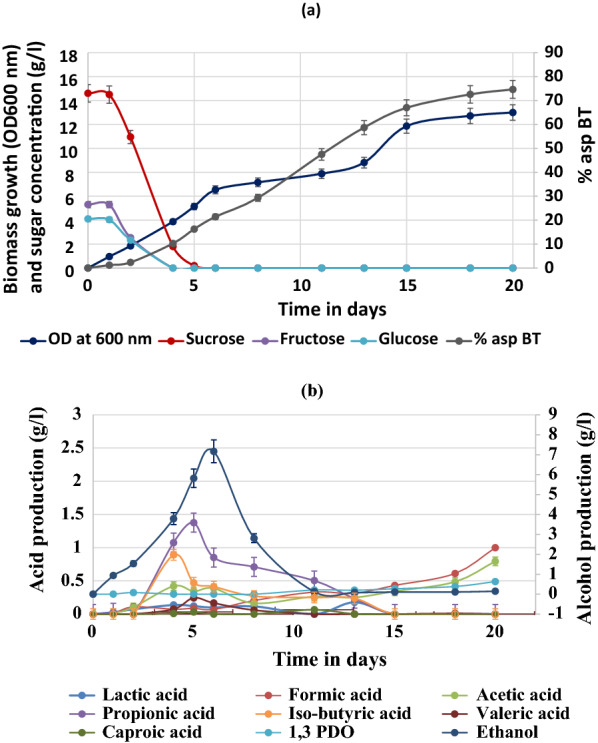
Kinetics of asphaltene biotransformation by growing cell consortium in a 1.5 l bioreactor using sucrose as a primary carbon source. **a** Biomass growth, sugar uptake and percentage asphaltene biotransformation profile. **b** Ethanol and acid formation profile during asphaltene biotransformation

Figure 4 shows the average data obtained from two reactor vessels run in parallel using a single Multifors 2 controller and the error bars correspond to the deviation observed in two reactors. The growth data obtained from the run indicated a diauxic growth of the consortium with a minimum lag phase and the first stationary phase kicking in after day 5 when the OD_600_ reached a value of 6.5 (Fig. 4a**)**. During the first exponential phase, sucrose and its thermal degradation products (glucose and fructose) were rapidly utilized and the fermentation products such as ethanol (6–10%, v/v and acids (acetic acid, butyric acid, lactic acid and propionic acid) were released (Fig. 4b). The first stationary phase was observed from day 6 and lasted for 2 days during which complete exhaustion of sucrose, glucose and fructose took place and microbes adapted to and started to consume the available carbon sources like ethanol and the low molecular weight acids. The secondary exponential phase started from day 13 and lasted till day 15 in which the OD_600_ increased to 12. This phase also marked the exhaustion of the fermentative products accumulated during the first exponential phase, i.e., ethanol and other acids. A sigmoidal curve for biotransformation of asphaltene was also observed throughout the process and at the end of the batch, about 75% biotransformation was obtained (Fig. 4a). The initial concentration of asphaltene used in the reactor was 2.50 g/l and the amount recovered after 3 weeks was 0.625 g/l with respect to the control.

### Enzyme production during asphaltene biotransformation

The results of enzyme assays are shown in Table 3. The presence of extracellular dioxygenases like catechol 2,3 dioxygenase and catechol 1,2 dioxygenases, laccases and peroxidases like lignin peroxidase and manganese peroxidase was observed in the culture medium (Table 3). The dioxygenases have the property of incorporation of oxygen into the aromatic substrates and thus appear to do so with asphaltene and open the aromatic rings of asphaltene for further action by other enzymes. Laccase and H_2_O_2_ dependent enzymes were also detected in the culture broth suggesting production of low molecular weight aromatics that can induce these enzymes.

**Table 3:** Enzyme production during asphaltene biotransformation

Enzyme assay	Result
Indole Indigo assay	Negative
Catechol 1,2 dioxygenase assay	Positive
Catechol 2,3 dioxygenase assay	Positive
Laccase	Positive
Lignin peroxidase	Positive
Manganese peroxidase assay	Positive

### Biosurfactant production by members of consortium

**Fig. 5:**
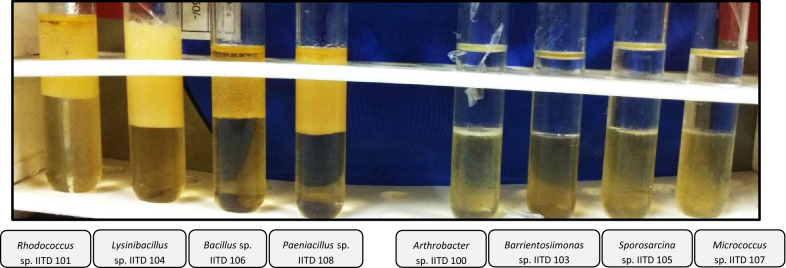
E_24_ assay for determination of biosurfactant production by members of consortium

Solution of 10% SDS and cell free supernatants of *Rhodococcus* sp. IITD 101, *Lysinibacillus* sp. IITD 104, *Bacillus* sp. IITD 106 and *Paenibacillus* sp. IITD 108 resulted in collapse of drop of supernatant within a minute of its addition to oil coated wells and thus these cultures were scored positive for the presence of the biosurfactant. The supernatants of the rest of the members of the consortium gave negative results. The results were consistent with oil displacement assay and emulsion index assay (Fig. 5) where in the solution of 10% SDS and the supernatants from the above-mentioned strains could displace the thin layer of oil (in oil displacement assay) and also resulted in the formation of stable emulsion when mixed with petrol (in emulsion index assay) (Table 4). Supernatants from the rest of the members and uninoculated medium gave negative results in these assays. Microplate assay was also scored positive for *Lysinibacillus* sp. IITD 104 and *Bacillus* sp. IITD 106.

**Table 4:** Results of biosurfactant production assays with different members of the consortium

Strain	Drop collapse assay	Microplate assay	Oil displacement assay	Cooper Goldberg assay	Surface tension reduction below 45 mN/m
*Arthrobacter* sp. IITD 100	–	–	–	–	–
*Arthrobacter* sp. IITD 101	–	–	–	–	–
*Rhodococcus* sp. IITD 102	Positive	–	Positive	Positive	Positive
*Barrientosiimonas* sp. IITD 103	–	–	–	–	–
*Lysinibacillus* sp. IITD 104	Positive	Positive	Positive	Positive	Positive
*Sporosarcina* sp. IITD 105	–	–	–	–	–
*Bacillus* sp. IITD 106	Positive	Positive	Positive	Positive	Positive
*Micrococcus* sp. IITD 107	–	–	–	–	–
*Paenibacillus* sp. IITD 108	Positive	–	Positive	Positive	Positive

Biosurfactant production by *Rhodococcus* sp. IITD 101, *Lysinibacillus* sp. IITD 104, *Bacillus* sp. IITD 106 and *Paenibacillus* sp. IITD 108 was confirmed as these strains could reduce the surface tension of the medium from 58.89 mN/m to 31.45 mN/m, 30.41 mN/m, 27.29 mN/m and 28.43 mN/m, respectively. The surface tension of double distilled water was found to be 71.95 mN/m and that of uninoculated medium was found to be 58.89 mN/m.

### Effect of biosurfactant on asphaltene present in model oil

**Fig. 6:**
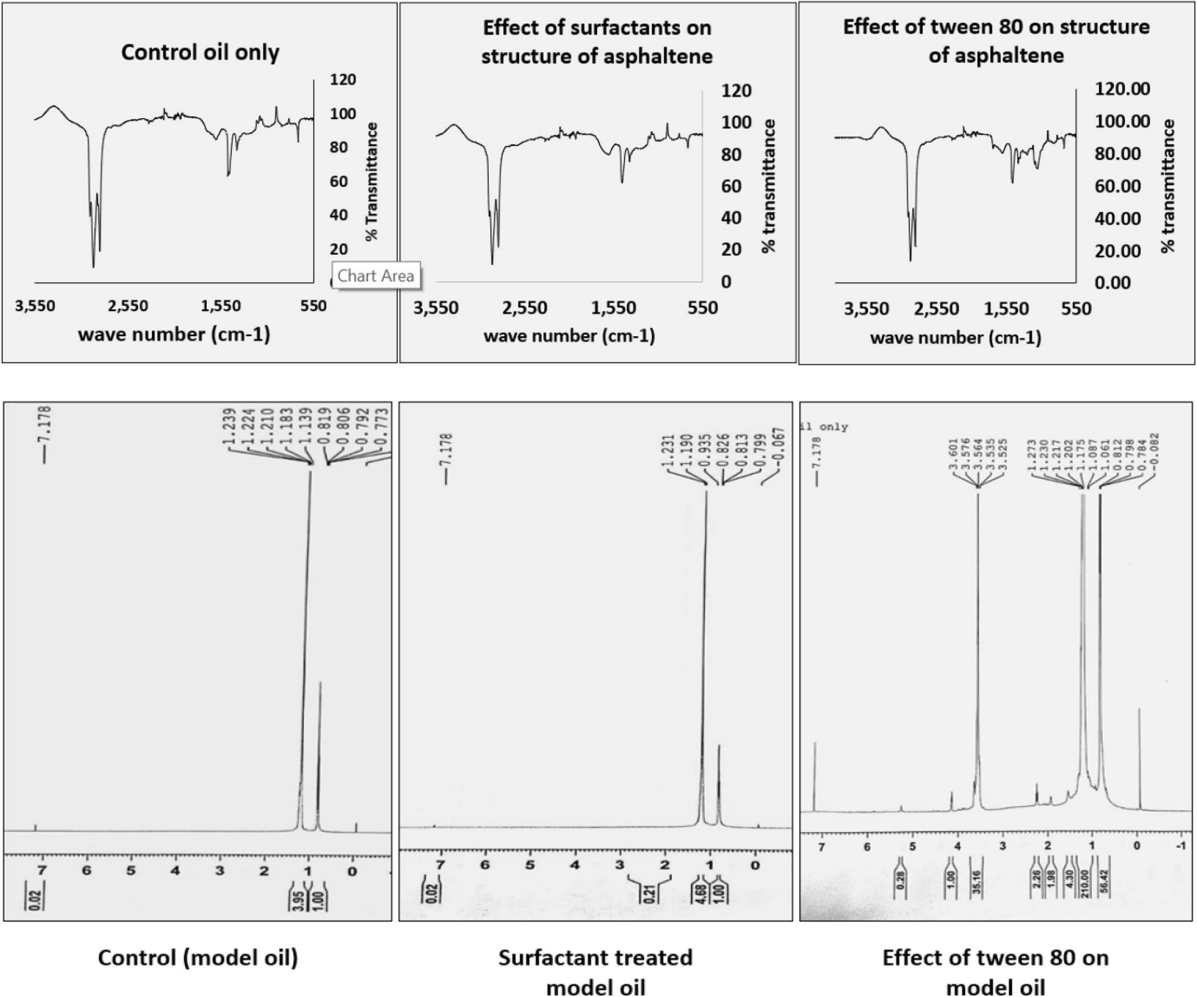
Effect of biosurfactants on the structure of asphaltene

Biosurfactant mixture added to the model oil (without the addition of the cells) did not result in any significant biotransformation. Only 4–5% of asphaltene was biotransformed in flasks where biosurfactant mixture was added to the model oil. On the other hand, in flasks where tween 80 was added, around 15–20% asphaltene biotransformation was observed. FT-IR and NMR studies also showed that the spectrum of the control asphaltene (harvested from flask in which only model oil was present) was exactly similar to that of the asphaltene in which biosurfactant mixture was added showing that the addition of biosurfactant did not have any effect on the structure of asphaltene (Fig. 6). On the other hand, quite a few changes were observed in the FT-IR and NMR spectra of asphaltene obtained from flask in which tween 80 was added suggesting that tween 80 effected the structure of asphaltene. This experiment led us to conclude that the biosurfactants produced by the members of the consortium during asphaltene biotransformation only help in reducing the interfacial tension between oil phase and aqueous phase and therefore promote mass transfer between the two phases and assist in biotransformation of asphaltene.

### Upgradation of heavy Maya crude oil

**Fig. 7:**
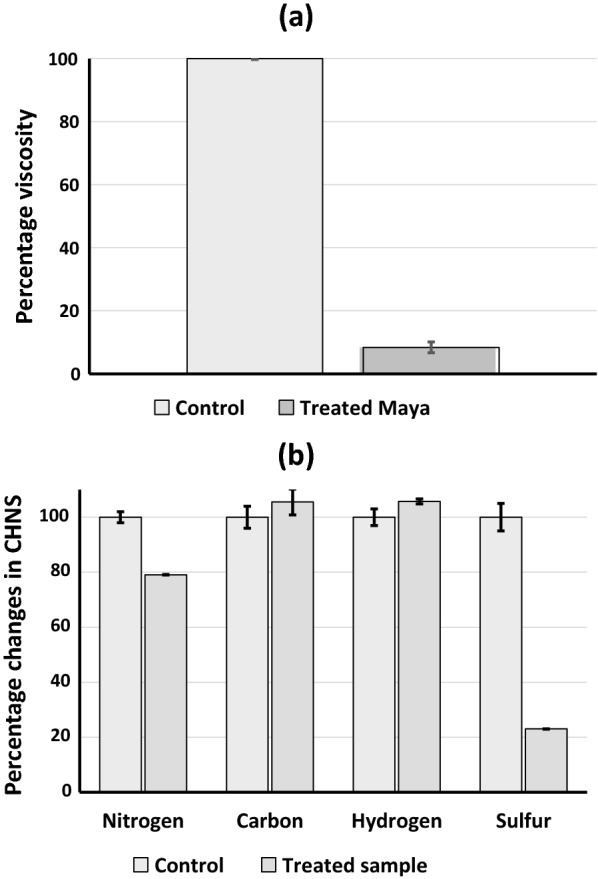
Viscosity reduction of heavy crude oil by the microbial consortium. **a** Percentage reduction in viscosity of Maya heavy crude oil due to upgradation by microbial consortium. **b** Elemental changes in Maya crude oil composition due to upgradation by microbial consortium

Viscosity measurements of the treated Maya crude oil revealed an average viscosity reduction of 91% by the consortium. Apparent viscosity of control was around 4.5 Pas when sheared at a rate of 200 s^−1^ while that of the treated samples was around 0.37 Pas (Fig. 7a). After upgradation by the consortium, major decrease was seen in the level of S and N and no decrease was observed in C and H. Seventy eight percent decrease was observed in the S content and 22% decrease was observed in the N content (Fig. 7b). Upgradation of heavy oil was also found to result in biotransformation of 60% of asphaltenes present in it.

## Discussion

In the present study a bacterial consortium was isolated which could bring about biotransformation of asphaltene and resulted in reduction of viscosity of the heavy crude oil. The consortium consisted of nine bacteria including Gram- negative and Gram-positive bacteria and was used to biodegrade and biotransform asphaltene in lab scale experiments. Out of the nine bacteria, with the exception of *Barrientosiimonas* sp., all other genera have been reported for polyaromatic hydrocarbon or crude oil biodegradation (Aoshima et al. 2006; Kawo and Bacha 2016; Küce et al. 2015; Li et al. 2018; Manchola and Dussán 2014; Shibulal et al. 2017; Shibulal et al. 2018). *Arthrobacter, Lysinibacillus, Bacillus* and *Micrococcus* have been reported for asphaltene degradation as well (Ali et al. 2012; Jahromi et al. 2014; Kuhad and Gupta 2009). The remaining bacterial genera such as *Sporosarcina* and *Paenibacillus* have not been reported for asphaltene biodegradation or biotransformation. While a number of individual bacterial genera have been reported to degrade asphaltene such as *Pseudomonas* sp. (Munawar et al. 2012), *Bacillus*, *Micrococcus, Proteus*, *Penicillium*, *Aspergillus* and *Rhizopus* (Okerentugba and OU 2003) etc., only few studies have reported asphaltene degradation by a bacterial consortium. Pineda-Flores et al. (2004) discovered a microbial consortium (*Bacillus*, *Brevibacillus, Corynebacterium*, and *Staphylococcus*) from Maya crude oil, which used asphaltene as a carbon and energy source (Pineda-Flores et al. 2004). This indicated the presence of asphaltene degrading microbes in the crude oil itself. Similarly, Ali et al. (2012) found three halotolerant species from oil polluted water samples namely, *Bacillus*, *Pseudomonas aeruginosa* and *Micrococcus*, which were also capable of using asphaltene as sole source of carbon and energy (Ali et al. 2012). Tavassoli et al. (2012) also found similar results with bacteria like *Pseudomonas*, *Bacillus licheniformis*, *Bacillus lentus*, *Bacillus cereus*, and *Bacillus firmus* (Tavassoli et al. 2012). In a recent study *Bacillus subtilis* and *B. licheniformis* were used for the biodegradation of heavy crude oil that led to enhanced oil recovery (Al-Sayegh et al. 2015; Al-Wahaibi et al. 2016). However, these reports on heavy oil biodegradation did not report specific decrease in the asphaltene content.

Vazquez-Duhalt and coworkers demonstrated 13.2% mineralization of asphaltene by a fungal strain *Neosartorya fischeri* (Uribe‐Alvarez et al. 2011). The authors debated that the surfactants produced by the microorganisms could open up the asphaltene structure leading to misinterpretation of the results. Using *N. fischeri,* the authors reported, for the first time, a decrease in asphaltene and concomitant release of carbon dioxide. Similarly, a halotolerant biosurfactant producer *P. aeruginosa* asph2 was shown to degrade 55% asphaltene in 21 days at 150 rpm and 30 °C (Ali et al. 2014). A major drawback in this study was the estimation of asphaltene using gravimetric method which resulted in its overestimation (Ali et al. 2014).

Both the gravimetric and the spectrophotometric methods (Jahromi et al. 2014) used in literature for determination of asphaltene cannot exactly determine the amount of asphaltene degraded or biotransformed. Using gravimetric methods, an over-estimation of the degraded asphaltene results as the loss in the weight could be due to disruption of the asphaltenic matrix rather than asphaltene itself. This has been attributed to production of biosurfactants which can result in release of entrapped hydrocarbons. On the other hand, current spectrophotometric methods can lead to overestimation of total asphaltene degraded because the degradation products or other microbial metabolites could be soluble in toluene and can therefore alter the exact estimation. Therefore, the method developed in the present study, both for extraction and accurate estimation of residual asphaltene, is novel and can be reliably used for estimation purpose. Using this method, an average decrease of about 73% of asphaltene was estimated in shake flask level experiments.

The isolated consortium was also used to carry out asphaltene biotransformation at a 1.5 l bench-scale bioreactor level under controlled conditions and 75% transformation achieved. While there are studies on asphaltene biodegradation performed in a bioreactor (Tavassoli et al. 2012), but to the best of our knowledge, no studies have been reported on biotransformation of asphaltene. A secondary log phase was observed when sucrose was over and the fermentation products such as acids and ethanol were used for growth. It is likely that some of the members of the bacterial consortium such as *Rhodococcus* sp., *Micrococcus* sp., *Lysinibacillus* sp. and *Arthrobacter* sp. would have taken off. There are reports of *Rhodococcus erythropolis* KA2-5-1which can grow on ethanol as carbon source and desulfurize Dibenzothiophene (DBT) efficiently. Similarly, *Lysinibacillus sphaericus* has been reported to grow on acetate as carbon source (Gomez-Garzon et al. 2017). Biodesulfurization of DBT has been shown by the strain *L. sphaericus* DMT-7 (Bahuguna et al. 2011). Further, *Arthrobacter* sp. Rue61a has been shown to utilize various carbon sources including ethanol (0.5 and 1%) and acetate (1 mM and 3 mM) (Niewerth et al. 2012). Another bacterium, *Micrococcus luteus* has been shown to utilize lactate as carbon source (Hiraishi and Komagata 1989).

In order to understand the biotransformation process, it was important to determine the structure of asphaltene. Multiple methods such as ^13^C-NMR, ^1^H-NMR, FT-IR, LC–MS and elemental analysis of asphaltene were used and a representative structure for asphaltene from Maya crude oil has been predicted. Using similar analytical methods, the structure of asphaltene has been recently reported from Golden Lane of Mexico oil and the molecular weight predicted to be 1039 g/mol (Ruiz-Morales et al. 2020). The molecular weight in the present study, however, was found to be 438–627 g/mol (Additional file 1: Fig. S6).

Biotransformation of asphaltene by the bacterial consortium resulted in formation of oxygenates and other metabolites which were lower in molecular weight, as determined with ^1^H-NMR and FT-IR. Several oxidized metabolites, were also detected by Vazquez-Duhalt and coworkers (Uribe‐Alvarez et al. 2011) and these were hydroxyphenylacetic acid, 9-nitroso carbazole, thianaphthene-2-carboxylic acid, and hydroxypyrenedione etc.

A decrease in S and N content of asphaltene (due to its biotransformation) and model oil (due to its upgradation) was observed without affecting the hydrocarbon content suggesting that the consortium biotransformed asphaltene. It is likely that the removed S and N from asphaltene were used for growth by the bacteria. The consortium also facilitated incorporation of oxygen into the structure of asphaltene. However, differential patterns of heteroatom losses were observed in different experiments (growing cell and resting cell asphaltene biotransformation and oil upgradation experiments) which could be explained in terms of the nature of the experiment and the compound of interest. In growing cell experiment, enzymes production occurs during the growth of the consortium in presence of asphaltenes, these enzymes act on asphaltene and remove the hetero atom content of these asphaltenes to a higher amount. However, in resting cell experiment, cells are prepared by growing them separately, without adding asphaltene and are then added to flasks containing model oil. Asphaltene induced enzymes will not be produced during preparation of resting cells. The cells when added to the flasks containing model oil cannot grow further because of very high density, and therefore no further enzyme production takes place. Hence, resting cells may lack some key enzymes and therefore show reduced asphaltene biotransformation and differential removal of heteroatoms. On the other hand, asphaltene in Maya crude oil is present in stable equilibrium with resins and their accessibility to the biotransforming enzymes could be limited or sterically hindered by resins. And since in crude oil aliphatic and aromatic fractions may also contain heteroatoms, a fraction of enzymes could be utilized in their degradation or removal. Therefore, exhibiting a different pattern of heteroatom degradation in model oil.

Four out of nine members of the consortium were found to be capable of producing biosurfactants. It was also established that the biosurfactants produced at the concentration in the experiments did not have a direct effect on the structure of asphaltene, they only reduced the mass transfer limitations between the two phases and therefore, increased the access of model oil to biotransforming microbes and enzymes.

In this study, upgradation of the Maya crude oil was also demonstrated using the bacterial consortium. About 91% decrease in the viscosity of the crude oil was observed within 2 weeks at an oil: water ratio of 1:9. In the past, a maximum of 88.7% decrease in asphaltene was reported by a strain of *Daedaleopsis* in 30 days (Pourfakhraei et al. 2018). The studies were also restricted to bioremediation and no attempts were made to use the consortium for decreasing the viscosity of the heavy crude oil (Pourfakhraei et al. 2018). The only report showing that asphaltene degradation can actually result in viscosity reduction was by Lal and coworkers (12). The reported isolate, *Garciaella petrolearia*, was shown to decrease the viscosity anaerobically by 42 and 37% with and without molasses, respectively. This reduction occurred under anaerobic conditions after 30 days of inoculation (Lavania et al. 2012).

In the present study, it was clearly demonstrated that microbes may reduce the viscosity of the heavy crude oil by biotransformation of asphaltene. The advantage of biological methods over other methods is their specificity and feasibility to transform and not degrade asphaltene at ambient temperature and pressure, which will not affect the calorific value of the heavy crude oil.

## Supplementary Information

**Additional file 1**: **Fig. S1**. Microbial strains isolated by enrichment culture method. **Fig. S2** (a) Growth profile of consortium in seven weeks growing cell experiment with asphaltene as primary carbon source, (b) three weeks resting cell experiment. Experiments were performed in triplicates. **Fig. S3** Biomass growth (a) and acid and alcohol production (b) during hexadecane biodegradation by microbial consortium, biomass growth and sucrose utilization (c) and acid and alcohol production (d) during hexadecane biodegradation using medium supplemented with sucrose and biomass growth (e) and acid and alcohol production (f) during biotransformation of crystalline asphaltene. **Fig. S4** Biomass growth and sucrose utilization (a) and acid and alcohol production (b) during asphaltene biotransformation when ammonium oxalate was replaced by ammonium chloride in BSM and supplemented with sucrose, biomass growth (c) and acid and alcohol production (d) during asphaltene biotransformation experiment with ammonium oxalate as carbon source, biomass growth and sucrose utilization (e) and acid and alcohol production (f) during biodegradation of heptamethylnonane when sucrose was added to the culture medium. **Fig. S5** Biomass growth and sucrose consumption (a) acid and alcohol production (b) during asphaltene biotransformation using heptamethylnonane as carrier phase. **Fig. S6** Representative structure of asphaltene used in the study.

## Data Availability

All data generated or analysed during this study are included in this published article [and its Additional files].
