# Supplementary material for: Asphaltene biotransformation for heavy oil upgradation
Source: AMB Express. 2021 Sep 7;11:127. doi: 10.1186/s13568-021-01285-7 (PMC8423941; doi:10.1186/s13568-021-01285-7)
Supplement: Supplementary file 1 — Additional file 1: Fig. S1. Microbial strains isolated by enrichment culture method. Fig. S2 (a) Growth profile of consortium in seven weeks growing cell experiment with asphaltene as primary carbon source, (b) three weeks resting cell experiment. Experiments were performed in triplicates. Fig. S3 Biomass growth (a) and acid and alcohol production (b) during hexadecane biodegradation by microbial consortium, biomass growth and sucrose utilization (c) and acid and alcohol production (d) during hexadecane biodegradation using medium supplemented with sucrose and biomass growth (e) and acid and alcohol production (f) during biotransformation of crystalline asphaltene. Fig. S4 Biomass growth and sucrose utilization (a) and acid and alcohol production (b) during asphaltene biotransformation when ammonium oxalate was replaced by ammonium chloride in BSM and supplemented with sucrose, biomass growth (c) and acid and alcohol production (d) during asphaltene biotransformation experiment with ammonium oxalate as carbon source, biomass growth and sucrose utilization (e) and acid and alcohol production (f) during biodegradation of heptamethylnonane when sucrose was added to the culture medium. Fig. S5 Biomass growth and sucrose consumption (a) acid and alcohol production (b) during asphaltene biotransformation using heptamethylnonane as carrier phase. Fig. S6 Representative structure of asphaltene used in the study. [file 13568_2021_1285_MOESM1_ESM.pptx]

## Slide 1
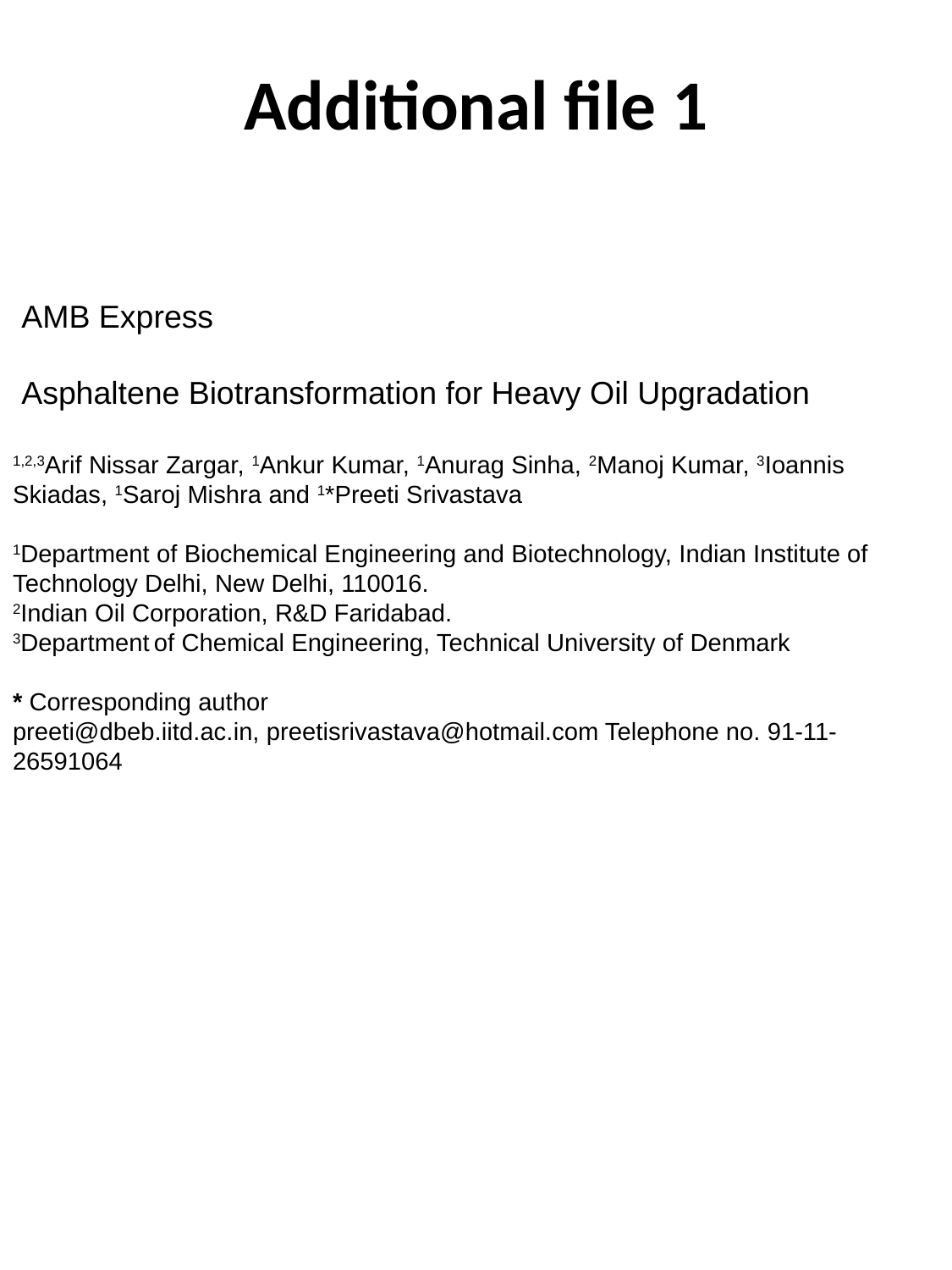

Additional file 1
 AMB Express
 Asphaltene Biotransformation for Heavy Oil Upgradation
1,2,3Arif Nissar Zargar, 1Ankur Kumar, 1Anurag Sinha, 2Manoj Kumar, 3Ioannis Skiadas, 1Saroj Mishra and 1*Preeti Srivastava
1Department of Biochemical Engineering and Biotechnology, Indian Institute of Technology Delhi, New Delhi, 110016.
2Indian Oil Corporation, R&D Faridabad.
3Department of Chemical Engineering, Technical University of Denmark
* Corresponding author
preeti@dbeb.iitd.ac.in, preetisrivastava@hotmail.com Telephone no. 91-11-26591064

## Slide 2
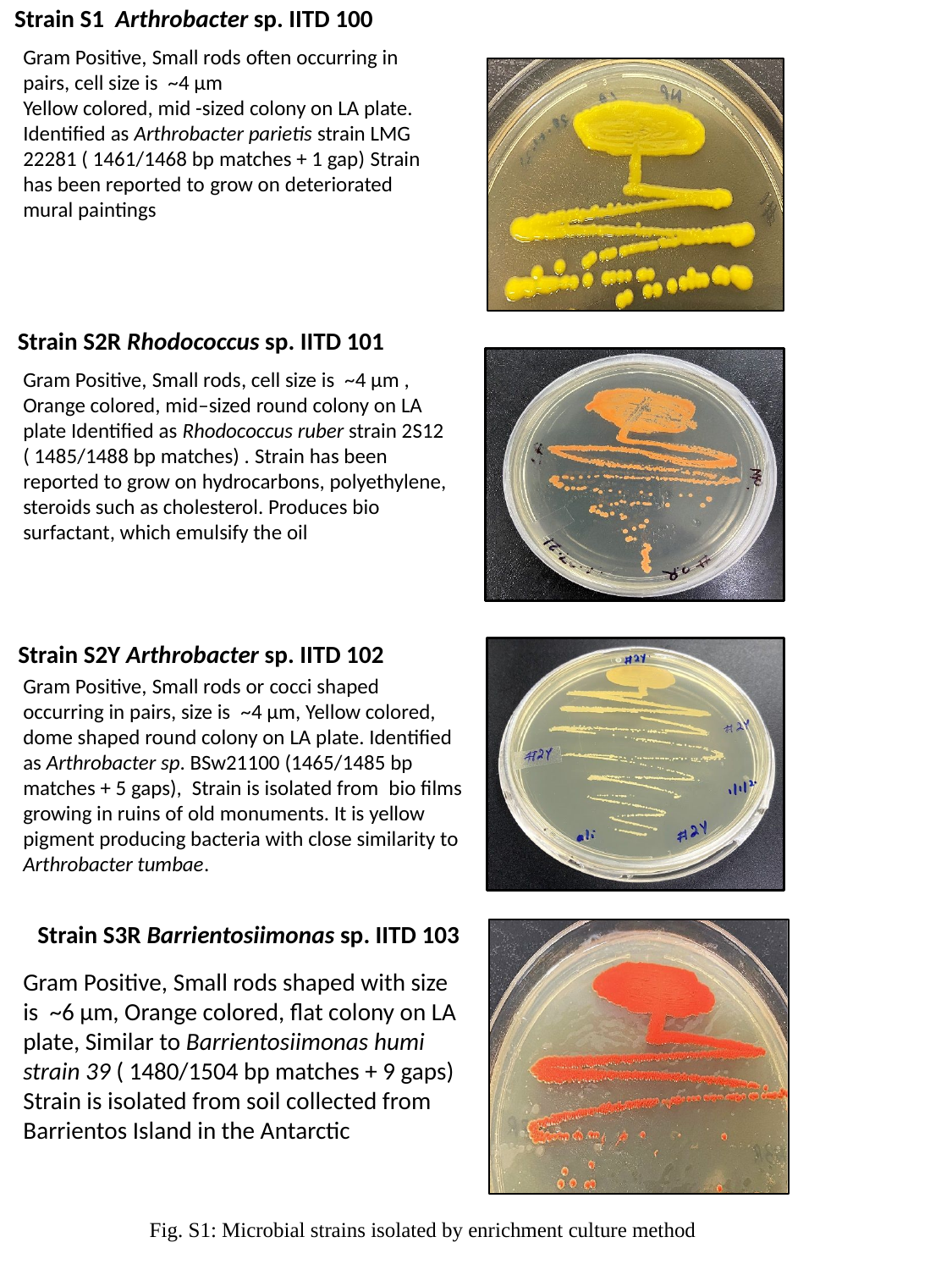

Strain S1 Arthrobacter sp. IITD 100
Gram Positive, Small rods often occurring in pairs, cell size is ~4 µm
Yellow colored, mid -sized colony on LA plate. Identified as Arthrobacter parietis strain LMG 22281 ( 1461/1468 bp matches + 1 gap) Strain has been reported to grow on deteriorated mural paintings
Strain S2R Rhodococcus sp. IITD 101
Gram Positive, Small rods, cell size is ~4 µm , Orange colored, mid–sized round colony on LA plate Identified as Rhodococcus ruber strain 2S12 ( 1485/1488 bp matches) . Strain has been reported to grow on hydrocarbons, polyethylene, steroids such as cholesterol. Produces bio surfactant, which emulsify the oil
Strain S2Y Arthrobacter sp. IITD 102
Gram Positive, Small rods or cocci shaped occurring in pairs, size is ~4 µm, Yellow colored, dome shaped round colony on LA plate. Identified as Arthrobacter sp. BSw21100 (1465/1485 bp matches + 5 gaps), Strain is isolated from bio films growing in ruins of old monuments. It is yellow pigment producing bacteria with close similarity to Arthrobacter tumbae.
Strain S3R Barrientosiimonas sp. IITD 103
Gram Positive, Small rods shaped with size is ~6 µm, Orange colored, flat colony on LA plate, Similar to Barrientosiimonas humi strain 39 ( 1480/1504 bp matches + 9 gaps)
Strain is isolated from soil collected from Barrientos Island in the Antarctic
Fig. S1: Microbial strains isolated by enrichment culture method

## Slide 3
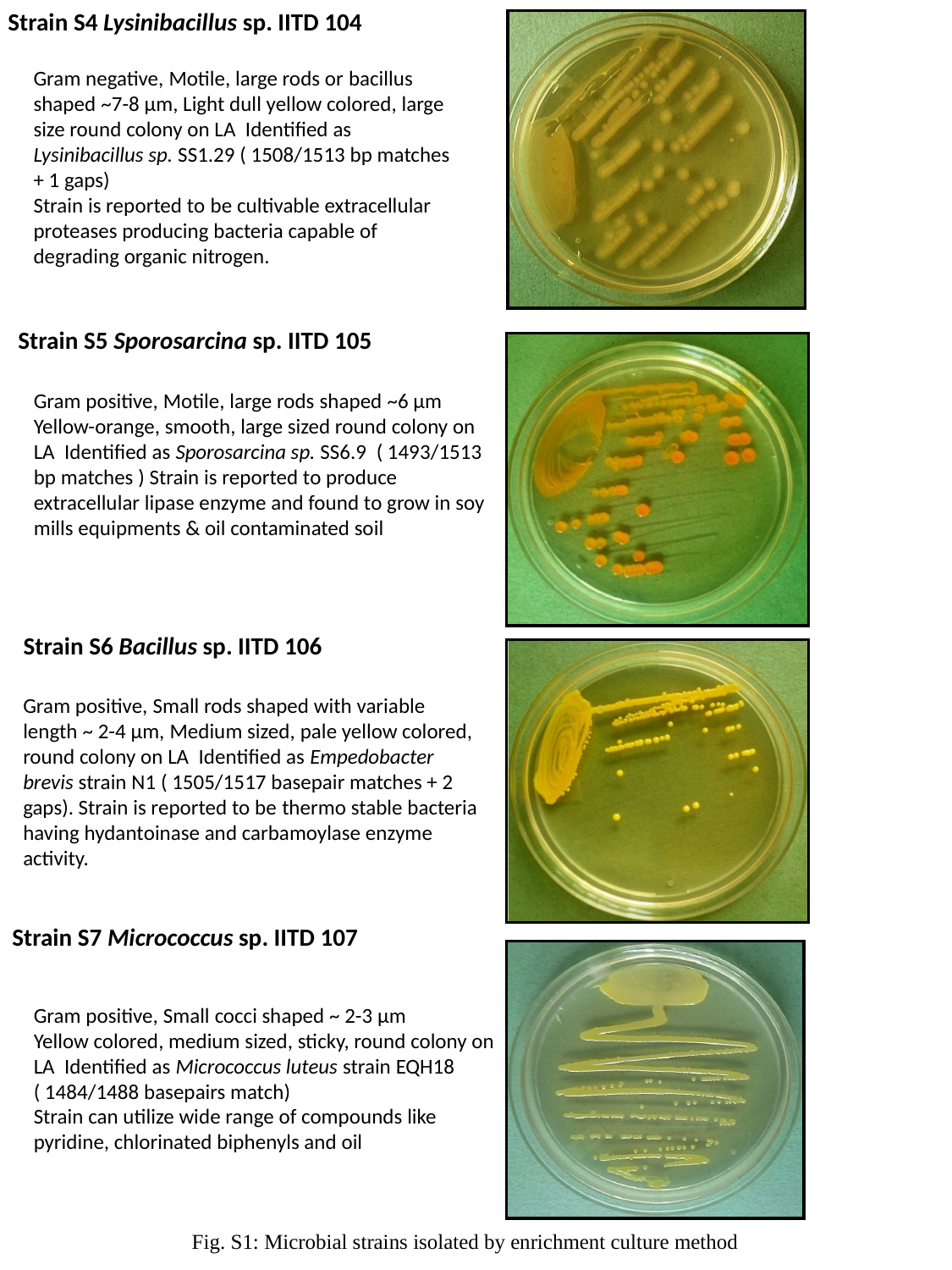

Strain S4 Lysinibacillus sp. IITD 104
Gram negative, Motile, large rods or bacillus shaped ~7-8 µm, Light dull yellow colored, large size round colony on LA Identified as Lysinibacillus sp. SS1.29 ( 1508/1513 bp matches + 1 gaps)
Strain is reported to be cultivable extracellular proteases producing bacteria capable of degrading organic nitrogen.
Strain S5 Sporosarcina sp. IITD 105
Gram positive, Motile, large rods shaped ~6 µm
Yellow-orange, smooth, large sized round colony on LA Identified as Sporosarcina sp. SS6.9  ( 1493/1513 bp matches ) Strain is reported to produce extracellular lipase enzyme and found to grow in soy mills equipments & oil contaminated soil
Strain S6 Bacillus sp. IITD 106
Gram positive, Small rods shaped with variable length ~ 2-4 µm, Medium sized, pale yellow colored, round colony on LA Identified as Empedobacter brevis strain N1 ( 1505/1517 basepair matches + 2 gaps). Strain is reported to be thermo stable bacteria having hydantoinase and carbamoylase enzyme activity.
Strain S7 Micrococcus sp. IITD 107
Gram positive, Small cocci shaped ~ 2-3 µm
Yellow colored, medium sized, sticky, round colony on LA Identified as Micrococcus luteus strain EQH18 ( 1484/1488 basepairs match)
Strain can utilize wide range of compounds like pyridine, chlorinated biphenyls and oil
Fig. S1: Microbial strains isolated by enrichment culture method

## Slide 4
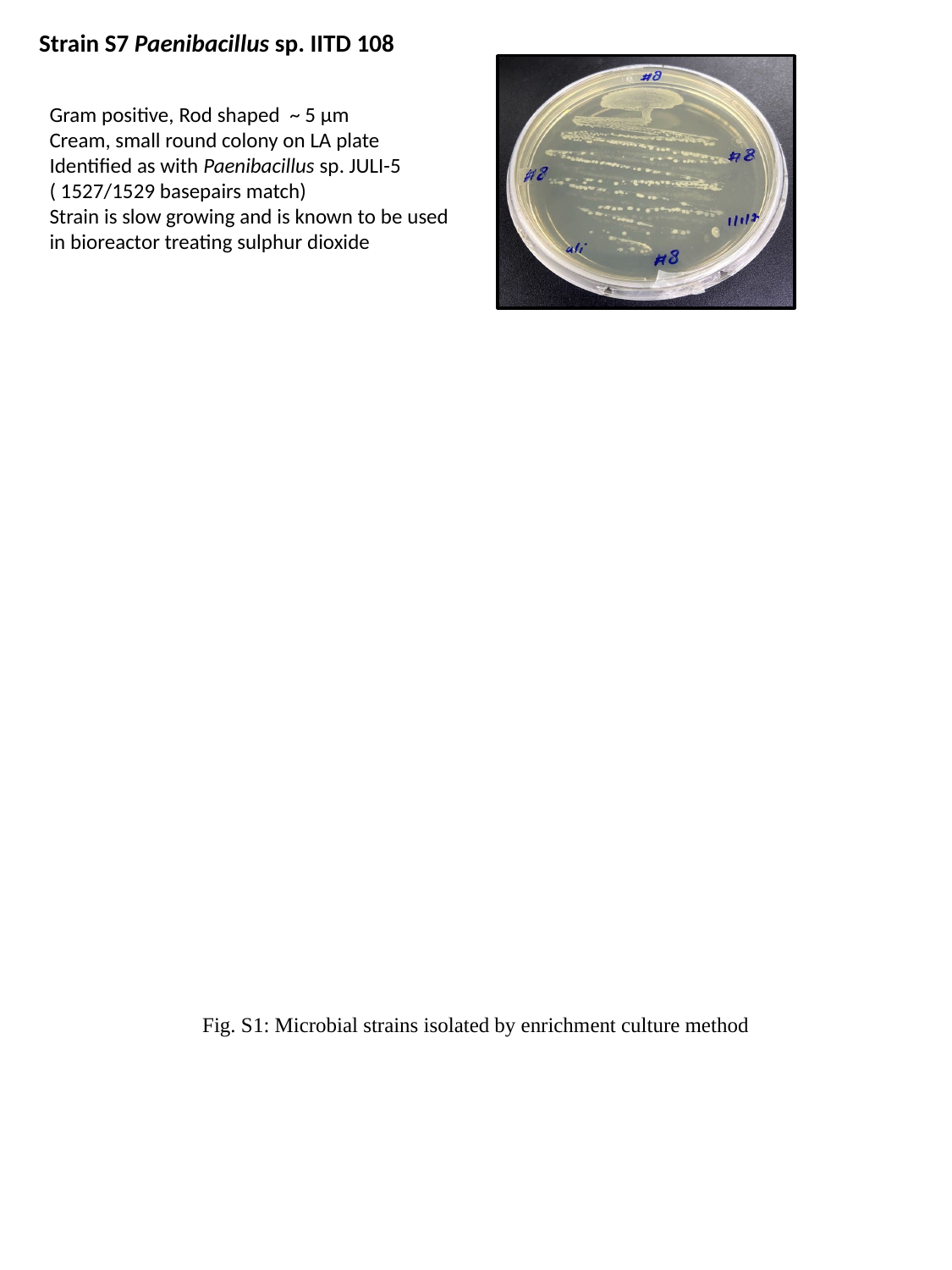

Strain S7 Paenibacillus sp. IITD 108
Gram positive, Rod shaped ~ 5 µm
Cream, small round colony on LA plate
Identified as with Paenibacillus sp. JULI-5  ( 1527/1529 basepairs match)
Strain is slow growing and is known to be used in bioreactor treating sulphur dioxide
Fig. S1: Microbial strains isolated by enrichment culture method

## Slide 5
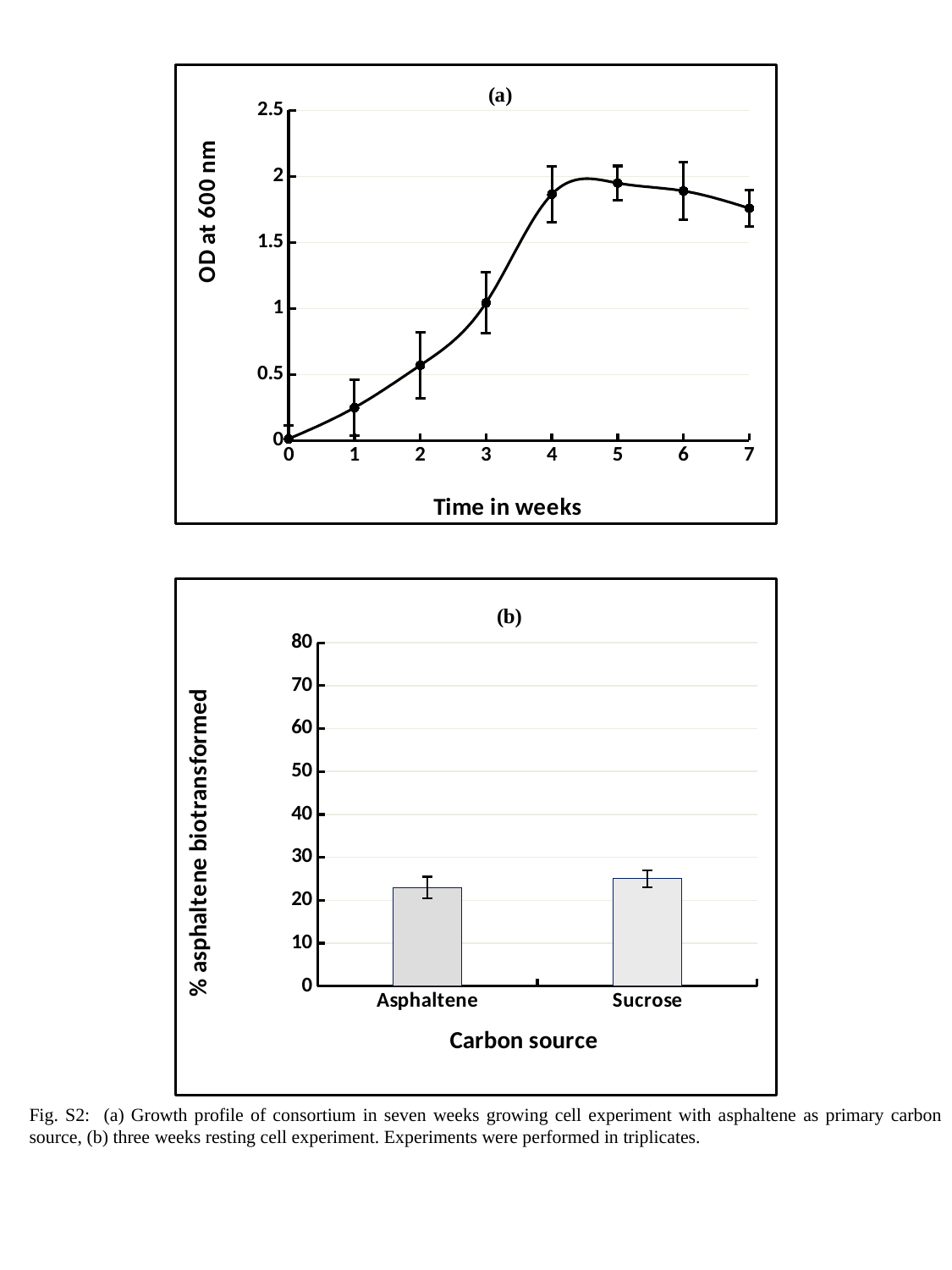

### Chart
| Category | |
|---|---|
### Chart
| Category | |
|---|---|
| Asphaltene | 23.0 |
| Sucrose | 25.0 |Fig. S2: (a) Growth profile of consortium in seven weeks growing cell experiment with asphaltene as primary carbon source, (b) three weeks resting cell experiment. Experiments were performed in triplicates.

## Slide 6
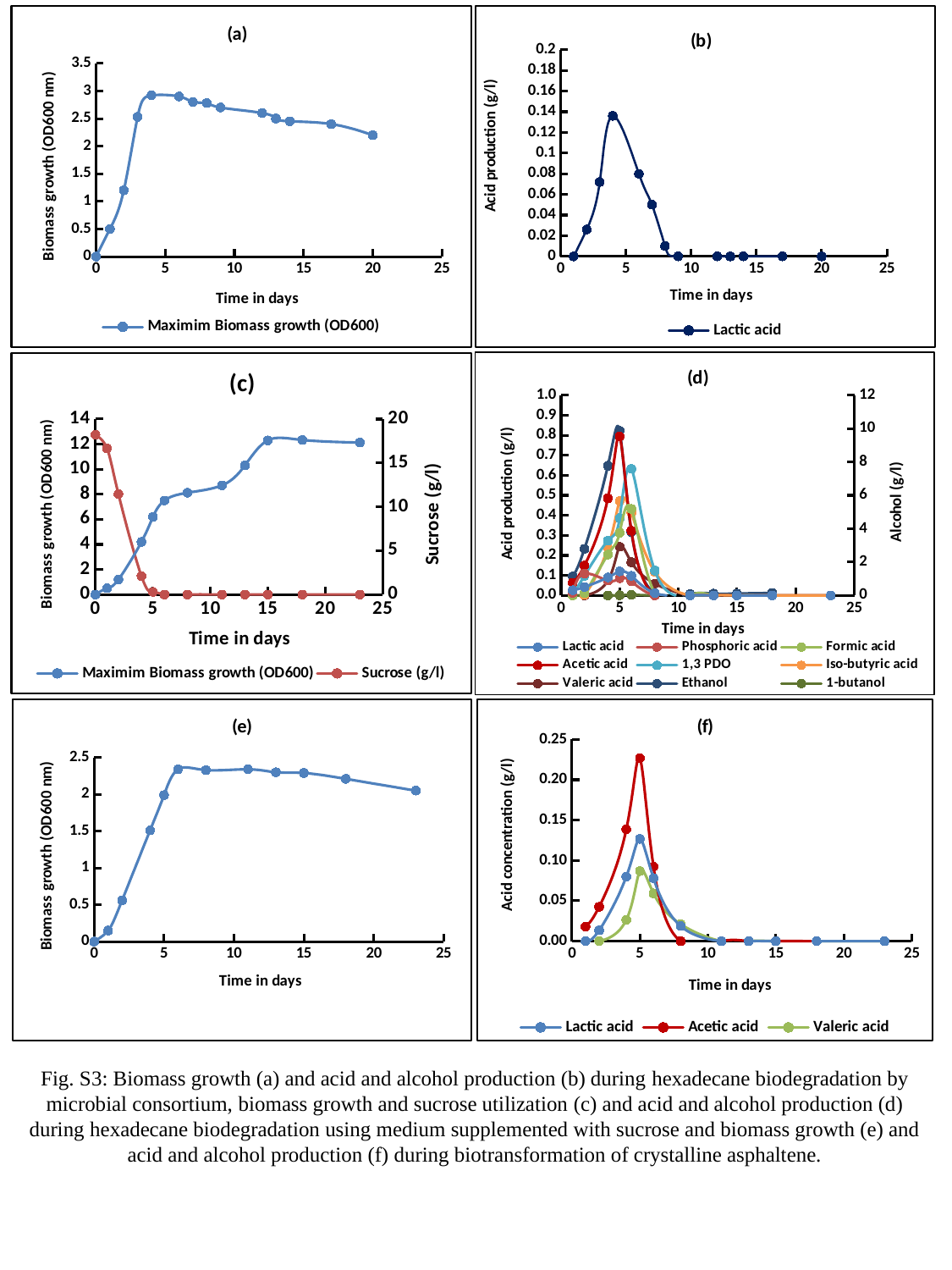

### Chart: (a)
| Category | Maximim Biomass growth (OD600) |
|---|---|
### Chart: (b)
| Category | Lactic acid |
|---|---|
### Chart: (d)
| Category | Lactic acid | Phosphoric acid | Formic acid | Acetic acid | 1,3 PDO | Iso-butyric acid | Valeric acid | Ethanol | 1-butanol |
|---|---|---|---|---|---|---|---|---|---|
### Chart: (c)
| Category | Maximim Biomass growth (OD600) | Sucrose (g/l) |
|---|---|---|
### Chart: (e)
| Category | Maximim Biomass growth (OD600) |
|---|---|
### Chart: (f)
| Category | Lactic acid | Acetic acid | Valeric acid |
|---|---|---|---|Fig. S3: Biomass growth (a) and acid and alcohol production (b) during hexadecane biodegradation by microbial consortium, biomass growth and sucrose utilization (c) and acid and alcohol production (d) during hexadecane biodegradation using medium supplemented with sucrose and biomass growth (e) and acid and alcohol production (f) during biotransformation of crystalline asphaltene.

## Slide 7
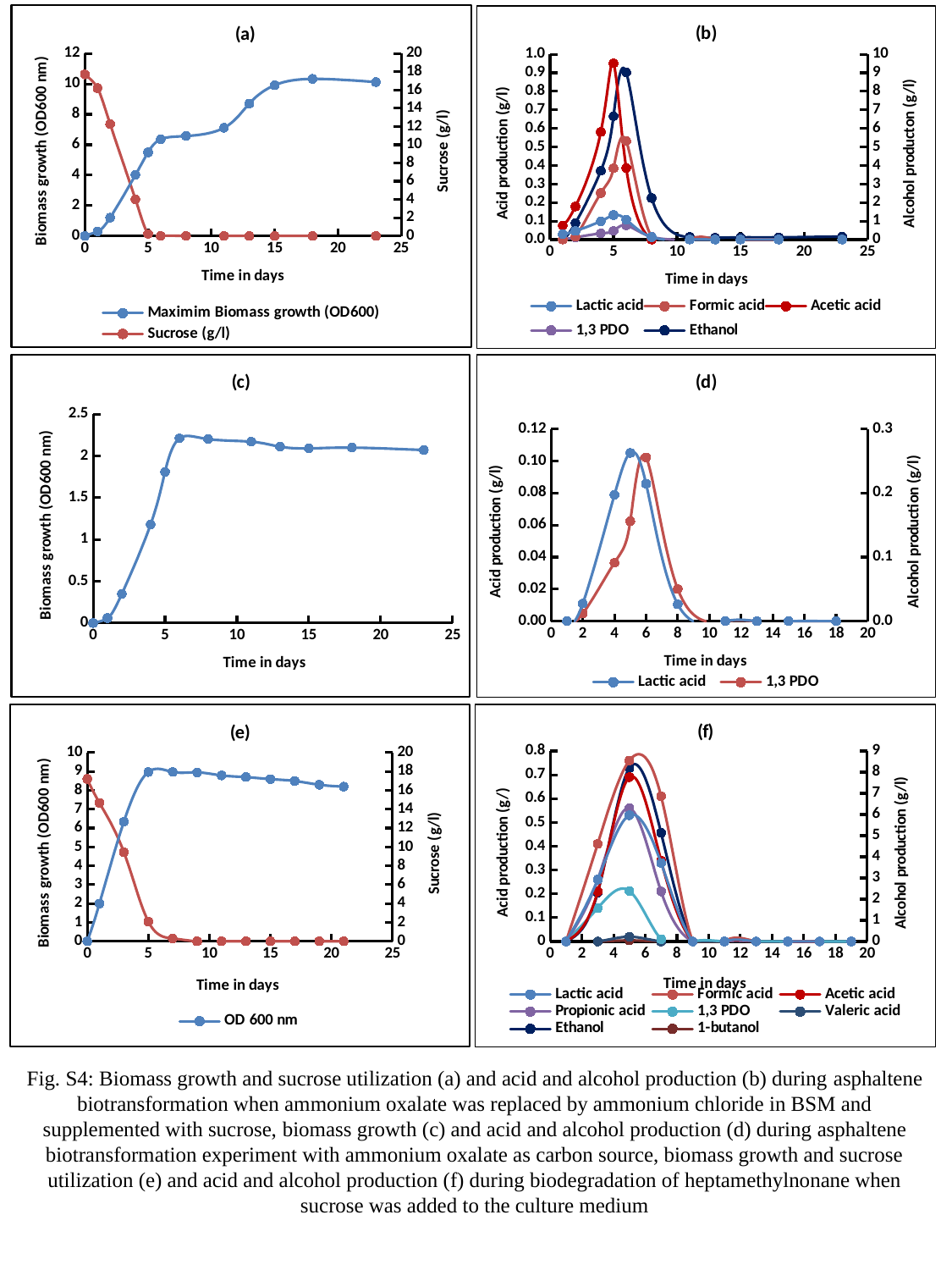

### Chart: (a)
| Category | Maximim Biomass growth (OD600) | Sucrose (g/l) |
|---|---|---|
### Chart: (b)
| Category | Lactic acid | Formic acid | Acetic acid | 1,3 PDO | Ethanol |
|---|---|---|---|---|---|
### Chart: (c)
| Category | Maximim Biomass growth (OD600) |
|---|---|
### Chart: (d)
| Category | Lactic acid | 1,3 PDO |
|---|---|---|
### Chart: (f)
| Category | Lactic acid | Formic acid | Acetic acid | Propionic acid | 1,3 PDO | Valeric acid | Ethanol | 1-butanol |
|---|---|---|---|---|---|---|---|---|
### Chart: (e)
| Category | OD 600 nm | Sucrose (g/l) |
|---|---|---|Fig. S4: Biomass growth and sucrose utilization (a) and acid and alcohol production (b) during asphaltene biotransformation when ammonium oxalate was replaced by ammonium chloride in BSM and supplemented with sucrose, biomass growth (c) and acid and alcohol production (d) during asphaltene biotransformation experiment with ammonium oxalate as carbon source, biomass growth and sucrose utilization (e) and acid and alcohol production (f) during biodegradation of heptamethylnonane when sucrose was added to the culture medium

## Slide 8
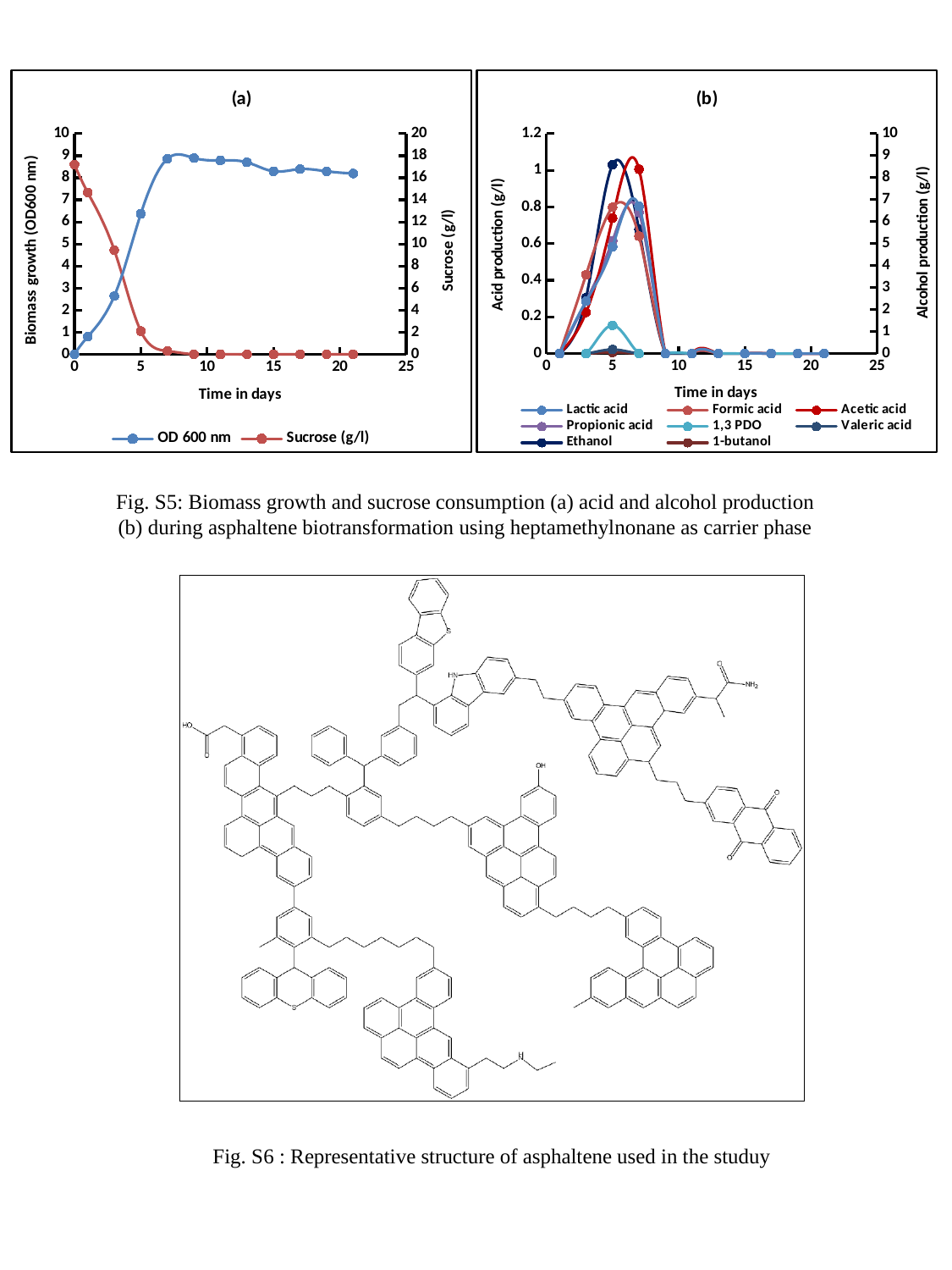

### Chart: (b)
| Category | Lactic acid | Formic acid | Acetic acid | Propionic acid | 1,3 PDO | Valeric acid | Ethanol | 1-butanol |
|---|---|---|---|---|---|---|---|---|
### Chart: (a)
| Category | OD 600 nm | Sucrose (g/l) |
|---|---|---|Fig. S5: Biomass growth and sucrose consumption (a) acid and alcohol production (b) during asphaltene biotransformation using heptamethylnonane as carrier phase
Fig. S6 : Representative structure of asphaltene used in the studuy
